# Integrative taxonomic reassessment of *Odontophrynus* populations in Argentina and phylogenetic relationships within Odontophrynidae (Anura)

**DOI:** 10.7717/peerj.6480

**Published:** 2019-02-25

**Authors:** Adolfo Ludovico Martino, Jonas Maximilian Dehling, Ulrich Sinsch

**Affiliations:** 1 Department of Ecology, National University of Rio Cuarto, Rio Cuarto (Cordoba), Argentina; 2 Department of Biology, Institute of Integrated Sciences, University of Koblenz-Landau, Koblenz, Germany

**Keywords:** Integrative taxonomy, Allozymes, Advertisement call, Species delimitation, *Macrogenioglottus*, *Odontophrynus*, Morphometry, 16S rRNA sequences, *Proceratophrys*

## Abstract

Amphibians are the most vulnerable vertebrates to biodiversity loss mediated by habitat destruction, climate change and diseases. Informed conservation management requires improving the taxonomy of anurans to assess reliably the species’ geographic range. The genus *Odontophrynus* that is geographically refined to Argentina, Bolivia, Brazil, Uruguay and Paraguay includes currently 12 nominal species with many populations of uncertain taxonomic assignment and subsequently unclear geographic ranges. In this study, we applied integrative taxonomic methods combining molecular (mitochondrial 16S gene), allozyme, morphological and bioacoustic data to delimit species of the genus *Odontophrynus* sampled from throughout Argentina where most species occur. The combined evidence demonstrates one case of cryptic diversity and another of overestimation of species richness. The populations referred to as *O. americanus* comprise at least three species. In contrast, *O. achalensis* and *O. barrioi* represent junior synonyms of the phenotypically plastic species *O. occidentalis*. We conclude that each of the four species occurring in Argentina inhabits medium to large areas. The Red List classification is currently “Least Concern”. We also propose a phylogenetic hypothesis for the genus and associated genera *Macrogenioglottus* and *Proceratophrys* (Odontophrynidae).

## Introduction

Distribution pattern of Neotropical amphibian diversity is not well understood because of incomplete information on taxonomy and distribution (Vieites et al., 2009; Winter et al., 2016). Yet amphibians are of high conservation concern, with almost 43% the currently known species being globally threatened and another 25% data deficient (Stuart et al., 2004). Taxonomic uncertainty stems partially from the prevalence of the morphospecies concept in most original descriptions of amphibian species (Frost, 2018). Morphological characters alone often fail to differentiate among closely related species due to the conservatism in the morphological evolution of anurans (Elmer, Dávila & Lougheed, 2007; Vences et al., 2010; Kaefer et al., 2013; Rojas et al., 2018). Advertisement calls act as powerful tools of premating isolation and can reveal morphologically cryptic species in sympatry, but in allopatry distinct species may give almost identical calls (Schneider & Sinsch, 2007; Köhler et al., 2017; Vacher et al., 2017). Delimiting species solely based on genetic distances obtained by barcoding approaches may inflate real species numbers by overestimating the taxonomic importance of intraspecific genetic structuring (Sukumaran & Knowles, 2017). Therefore, species delimitation should attempt to unite several lines of evidence to provide robust taxonomic hypotheses (Dayrat, 2005; Padial & De La Riva, 2010; Rojas et al., 2018).

South American anurans provide several examples for morphologically highly conserved genera in which integrative taxonomy led to reliable species delimitation and subsequent conservation priorities (De Magalhães et al., 2018; Fouquet et al., 2018; Von May, Lehr & Rabosky, 2018). The semi fossorial toads of the genus *Odontophrynus* pose a similar challenge because all original species descriptions are solely based on morphology and often too ambiguous for a reliable species distinction (Duméril & Bibron, 1841; Berg, 1896; Cei, Ruiz & Beçak, 1982; Di Tada et al., 1984; Cei, 1985; Martino & Sinsch, 2002; Rosset et al., 2006, 2007; Rosset, 2008; Caramaschi & Napoli, 2012; Rocha et al., 2017). Extant populations are currently assigned to 12 species that are placed into three phenetic groups based on overall morphological similarities, except for *O. salvatori* that is possibly a misplaced *Proceratophrys* species (Caramaschi, 1996; Amaro, Pavan & Rodrigues, 2009; Caramaschi & Napoli, 2012; Frost, 2018). The *O. americanus* group includes five nominal species (*O. americanus, O. cordobae, O. juquinha, O. lavillai* and *O. maisuma*), the *O. occidentalis* group three nominal species (*O. achalensis*, *O. barrioi* and *O. occidentalis*), and the *O. cultripes* group again three nominal species (*O. carvalhoi, O. cultripes* and *O. monachus*). *Odontophrynus* toads inhabit a latitudinal range of 5°S–41°S east of the Andes covering an altitudinal range from sea level to montane valleys of about 2,200 m above sea level (Turazzini, Taglioretti & Gomez, 2016; Santos-Silva et al., 2017).

The reliability of taxonomic assignment of populations to the currently recognized species is hampered by the overall similarity of external morphology, and assumed geographic ranges bear a high degree of uncertainty. Therefore, the status in the IUCN Red List of Threatened Species and resulting conservation needs are at least debatable, with seven species considered as “Least Concern,” one as “Vulnerable” and five as “Data Deficient” (IUCN, 2018). The three disjunct areas inhabited by the tetraploid *O. americanus* may indicate the presence of cryptic species (Rosset et al., 2006). The *Odontophrynus* sp. of the Sierra de San Luis may or not be conspecific with *O. achalensis* described from the Sierras de Cordoba (Di Tada et al., 1984). Diploid *O. americanus*-like populations reported from the vicinity of the disjunct *O. americanus* ranges have been recently described as three distinct species, *O. cordobae* in Central Argentina (Martino & Sinsch, 2002), *O. maisuma* in coastal Uruguay and Brazil (Rosset, 2008) and *O. juquinha* in Minas Gerais, Brazil (Rocha et al., 2017). It remains controversial, if diploids of the *O. americanus* group derived from tetraploids or tetraploids several times independently from diploids (Beçak & Beçak, 1974; Beçak, 2014). With respect to these issues and the validity of the phenetic groups within *Odontophrynus*, the most recent molecular phylogeny of *Odontophrynus* is inconclusive being based on only three of the 12 nominal taxa (Dias et al., 2013). Earlier phylogenies proposed by Amaro, Pavan & Rodrigues (2009), Pyron & Wiens (2011) as well as the recent one by Feng et al. (2017) agree in that Odontophrynidae is monophyletic and that *Macrogenioglottus* and *Odontophrynus* are sister taxa.

Consequently, a reliable taxonomic and geographic delimitation of *Odontophrynus* species requires an integrative approach critically evaluating information available on genetic differentiation and phenotypic plasticity. In this study we adopt the consensus protocol for integrative taxonomy to delimit species (Padial et al., 2010). Geographically, we focus on Argentina where most currently recognized *Odontophrynus* species and several populations of still undetermined taxonomic status occur. The character complexes included in the re-assessment of taxa are quantitative morphometrics, advertisement call features, allozyme differentiation and partial mitochondrial 16S rRNA sequences, all providing meaningful taxonomic information. Data refer to 34 populations, among them those at the type localities for reference. Sites of sympatry (*O. americanus/O. occidentalis, O. cordobae/O. occidentalis*) are contrasted with those in narrow contact zones (*O. americanus/O. cordobae, O. achalensis/O. occidentalis*) and sites of allopatry. Additional data on the molecular *Odontophrynus* diversity in Brazil are used for a broader phylogenetic view on Odontophrynidae (Amaro, Pavan & Rodrigues, 2009; Dias et al., 2013; Lyra, Haddad & De Azeredo-Espin, 2017). Specifically, we test the following hypotheses: (1) Phenotypic plasticity within and among *Odontophrynus* taxa is associated with corresponding genetic differentiation; (2) The phenetic groups within *Odontophrynus* represent distinct phylogenetic lineages; (3) The current Red List classification does not reflect the risks upon each species.

## Materials and Methods

### Study area and field sampling

We identified and sampled 34 local populations of toads pertaining to the genus *Odontophrynus* in Argentina (Table S1). The type localities of the nominal taxa *O. achalensis* Di Tada et al., 1984 (Pampa de Achala, Cordoba province), *O. barrioi* Cei, Ruiz, and Beçak, 1982 (Aguadita springs, Sierra de Famatina, La Rioja province), *O. cordobae* Martino & Sinsch, 2002 (Villa General Belgrano, Cordoba province) and *O. lavillai* Cei, 1985 (Villa de la Punta, Santiago del Estero province) were sampled to obtain topotypical individuals for taxonomic comparison. Unfortunately, the exact type localities of the most wide-spread species *O. americanus* (Duméril & Bibron, 1841) and *O. occidentalis* (Berg, 1896) are unknown because the original descriptions only state that the holotype *of O. americanus* was “sent from Buenos Aires” and that the holotype of *O. occidentalis* was collected in “Arroyo Agrio” in the Neuquén province (Frost, 2018). According to Lescure et al. (2002), the type locality of *O. americanus* is probably “rives du Rio Negro, Patagonie” in the Río Negro province (Argentina; D’Orbigny, 1847) at the southernmost part of the current geographical range. Still, populations of tetraploid *O. americanus* were readily distinguished from those of the diploid taxa by erythrocyte size (Rosset et al., 2006; Grenat, Salas & Martino, 2009). Populations of uncertain taxonomic assignment were tentatively referred to as *O.* cf. *achalensis* (Locality: La Carolina, San Luis province) or *O.* cf. *barrioi* (Localities: Aguada de Molle, Huerta de Guachi, San Juan province; Table S1). Material and data collected at the study sites were: (1) blood smears for ploidy assessment; (2) adult specimens for morphometric measurements, (3) records of advertisement calls, (4) muscle and liver homogenates for allozyme analyses and (5) alcohol preserved tissue for phylogenetic analyses (partial sequences of the mitochondrial 16S rRNA gene). The carcasses of specimens studied were deposited in museum collections; Table S1. The Córdoba Environment Agency (A.C.A.S.E.), Environmental Secretary of Córdoba Government (A01-2013), authorized our study and issued research and collecting permits.

### Morphological data

In a first step, presumed ploidy (diploid/tetraploid) was verified by measuring the erythrocyte size, which correlates with the DNA content. Smears of fresh blood were air-dried and light-microscopically examined at a magnification of 1,000 × using an OLYMPUS BX50 following the procedures described in Grenat, Salas & Martino (2009). Specimens were euthanized, tissues sampled and carcasses preserved in 4% formaldehyde. Use of vertebrate animals was approved by the Ethics Committee Comité de Ética de la Investigación (COEDI) of the Universidad Nacional de Rio Cuarto. (https://www.unrc.edu.ar/unrc/coedi/index.html). The investigation was conducted according to the state law “Protection and Conservation of Wild Fauna” (Argentina National Law N° 22.421) and the Ethical Committee of Investigation of the National University of Río Cuarto (N° 38/11). The external morphology of 256 specimens was described quantitatively by measuring 15 morphometric distances (to the nearest 0.1 mm; Martino & Sinsch, 2002): (1) Snout-vent length (SVL); (2) maximal head width (HW); (3) head length (HL); (4) snout-eye distance (SED); (5) internarinal distance (IND); (6) interocular distance (IOD); (7) eye-narinal distance (END); (8) rostronarinal distance (RND); (9) eye diameter (ED); (10) humerus length (HL); (11) length of 3rd finger (F3L); (12) femur length (FL); (13) tibia length (TL); (14) foot length (FOL); (15) length of 4th toe (T4L). All measurements were taken by the first author. Morphometric raw data are available in Dataset S1.

Each variable was standardized by subtracting the sample mean and dividing by the sample standard deviation. The matrix of standardized variables was subjected to a principal component analysis with a fixed number of three PCs extracted. By this means, we explored the morphometric variability independent of taxonomic assignment and reduced the information to statistically unrelated factors. PC1 represents size-related features, PC2 and PC3 shape-related ones. Separate PCAs were run on the taxa of the phenetic groups. Assignment of populations to a phenetic group was based on the advertisement call structure (*O. americanus*-group: simple pulsed calls; *O. occidentalis*-group: complex calls consisting of several pulse groups; Salas & Di Tada, 1994; Martino & Sinsch, 2002). The morphospace built by three PC-axes was used to evaluate partitioning among taxa. A discriminant analysis (Procedure: backward selection) with a priori taxon assignment was applied to quantify the partitioning of morphospace for each phenetic group. Removal of one or more morphometric variables is based on an *F*-to-remove test. If the least significant variable has an *F*-value less than 4.0, it will be removed from the model. We used the discriminant analyses to assess the magnitude of correct taxon classification for the individuals of each predefined group. Due to the partial sympatry among species of the *O. occidentalis* group we tested for clinal variation of PCs along latitudinal and altitudinal gradients by a multiple regression analysis (Procedure: backward selection at *F* = 4.0). Significance level was set to α = 0.05. All calculations were performed using the statistical package statgraphics centurion, version XVIII (Statpoint Inc., Warrenton, Virginia, USA).

### Bioacoustic data

Advertisement calls given by 302 individuals (series of 11–116 calls per individual) were recorded in the field using a DAT recorder Sony TCD-100^©^ with stereo microphone ECM-MS907 Sony^©^ and tapes TDK DA-RGX 60^©^ (Table 1). Ambient temperature (to the nearest 0.5 °C) was registered at the individual calling sites (usually shallow water near shore) immediately after recording. Short advertisement call series are available as Audio S1–S8. Whenever possible, specimens were collected to obtain tissue samples and for morphometric measurements. Oscillograms, sonograms and power spectra of the call series were prepared with the Medav Mosip 3000 Signal Processing System or the PC program Adobe Audition 1.0. Each call series was characterized by nine parameters which were measured in three calls per series (terminology and procedure according to Martino & Sinsch (2002) and Schneider & Sinsch (2007)): (1) call duration (ms); (2) number of pulse groups per call (*N*); (3) duration of pulse group (ms); (4) interval between pulse groups; (5) pulses per pulse group (*N*); (6) pulse duration (ms); (7) interpulse interval (ms); (8) pulse rate (pulses/s); (9) dominant frequency (Hz). Bioacoustic raw data are available in Dataset S2.

**Table 1:** Principal component analyses of morphometric and call data sets.

(A) Individuals of the *O. americanus*-group							
Morphometric variables with *N* = 151 observations	PC1	PC2	PC3	Call variables with *N* = 227 observations	PC1	PC2	PC3
	Eigenwert: 9.71 Variance explained: 64.7%	Eigenwert: 0.97 Variance explained: 6.5%	Eigenwert: 0.90 Variance explained: 6.0%		Eigenwert: 2.70 Variance explained: 45.0%	Eigenwert: 1.48 Variance explained: 24.7%	Eigenwert: 0.94 Variance explained: 15.6%
SVL	0.289	−0.129	0.025	Call duration	0.286	0.497	0.508
HW	0.295	−0.103	0.096	Pulses per call	−0.351	0.138	0.738
HL	0.227	−0.182	0.348	Pulse duration	0.454	0.352	−0.229
SED	0.226	−0.510	−0.067	Interpulse duration	0.486	−0.342	0.229
IND	0.221	0.067	−0.364	Pulse rate	−0.589	0.060	−0.147
IOD	0.165	−0.039	0.754	Dominant frequency	0.074	−0.700	0.266
END	0.258	−0.047	0.106				
RND	0.220	−0.463	−0.303				
ED	0.246	−0.269	−0.150				
HL	0.296	0.144	−0.080				
FL	0.278	0.216	−0.056				
TL	0.298	0.162	0.092				
FOL	0.289	0.218	−0.026				
F3L	0.264	0.371	−0.134				
T4L	0.261	0.324	−0.047				

(B) Individuals of the *O. occidentalis-*group							
Morphometric variables with *N* = 105 observations	PC1	PC2	PC3	Call variables with *N* = 75 observations	PC1	PC2	PC3
	Eigenwert: 10.53 Variance explained: 70.2%	Eigenwert: 0.79 Variance explained: 5.3%	Eigenwert: 0.69 Variance explained: 4.6%		Eigenwert: 3.41 Variance explained: 37.9%	Eigenwert: 1.99 Variance explained: 22.1%	Eigenwert: 1.62 Variance explained: 18.0%
SVL	0.286	−0.001	0.010	Call duration	0.397	−0.079	0.429
HW	0.291	−0.009	0.020	Pulse groups per call	0.390	−0.179	0.422
HL	0.222	−0.286	0.551	Pulse group duration	−0.236	0.473	0.248
SED	0.221	−0.650	−0.168	Interpulse group interval	0.059	0.612	0.134
IND	0.223	0.485	−0.381	Pulses per pulse group	−0.453	0.138	0.073
IOD	0.196	0.016	0.326	Pulse duration	−0.231	−0.117	0.600
END	0.255	−0.093	0.012	Interpulse duration	0.328	0.396	−0.342
RND	0.234	−0.328	−0.530	Pulse rate	−0.365	−0.384	−0.194
ED	0.247	−0.057	−0.270	Dominant frequency	0.362	−0.160	−0.196
HL	0.288	0.075	−0.036				
FL	0.272	0.237	0.099				
TL	0.289	0.045	0.166				
FOL	0.284	0.052	0.121				
F3L	0.270	0.236	−0.033				
T4L	0.270	0.135	0.094				

**Note:**

For details see text.

The arithmetic means of these call parameters were calculated for each series (=individual) and used for further analyses. Thus, the basic data set describing the advertisement calls of the populations studied consisted of 10 variables (nine call parameters and the corresponding ambient temperature) with *N* = 302 observations. As several call variables co-vary with ambient temperature, we calculated linear regression models of call parameter vs. temperature and used the standardized residuals to obtain a temperature-adjusted data set for further analysis. Analogous to the treatment of morphometric data, a principal component analysis was run on call data subsets of populations with homologous call structure (simple calls with six variables vs. complex calls with nine variables) to explore the bioacoustic differentiation among the taxa of each phenetic group. The three PCs explaining most of the variance were extracted to describe the sound space utilized by *Odontophrynus* and its partitioning among taxa. Moreover, a discriminant analysis (procedure: backward selection at *F* = 4.0) was applied on standardized call variables to quantify the partitioning of among-taxon sound space. We used the discriminant analyses to assess the magnitude of correct taxon classification for the individuals of each predefined group. Due to the partial sympatry among species of the *O. occidentalis* group we tested for clinal variation of PCs along latitudinal and altitudinal gradients by a multiple regression analysis (Procedure: backward selection at *F* = 4.0).

### Allozyme data

Liver samples were obtained from 147 individuals (Table S2). Samples were dissolved in one ml homogenate buffer (Tris–EDTA-NADP at pH 7.0) and stored at −65 °C until use. Aliquots of 0.3–3 μl liver homogenate were applied to commercial cellulose acetate plates (PHERO-cel, 5.7 × 14.0 cm) and submitted to a continuous horizontal electrophoresis (Hebert & Beaton, 1993). Buffer systems and duration of electrophoresis were 30–40 min at room temperature: (1) Tris–Glycine at pH 8.5 and constant 200 V; (2) Citric acid aminopropyl morpholine at pH 7.0 and constant 130 V. Following electrophoresis, each gel was stained using standard recipes (Murphy et al., 1996).

The allozyme pattern of liver tissue consisted of 10 enzyme systems controlled by a total of 14 putative loci: aspartate amino transferase (two loci, AAT, EC 2.6.1.1), carboxylesterase (1, EST, 3.1.1.1), glycerol-3-phosphate dehydrogenase (1, G3PD, 1.1.1.8), glucosephosphate isomerase (1, GPI, 5.3.1.9), isocitrate dehydrogenase (2, IDH, 1.1.1.42), lactate dehydrogenase (1, LDH, 1.1.1.27), malate dehydrogenase (2, MDH, 1.1.1.37), malic enzyme (1, ME, 1.1.1.40), 6-phosphogluconate dehydrogenase (1, 6PGD, 1.1.1.44), phosphoglucomutase (2, PGM, 2.7.5.1). Mitochondrial and cytoplasmatic loci were distinguished by prefixes (m/c), electromorphs (putative alleles) of each locus were designated alphabetically from cathode to anode. For reference, we used a sample of one *O. americanus* individual in each run.

Statistical analyses of data included the calculation of allele frequencies (Table S2) and Nei’s unbiased genetic distances (Nei, 1972). Distances >0.1 were considered indicative for differentiation at species level (Highton, 1999; Scillitani & Picariello, 2000). Calculation was performed using the program GENDIST of the Phylogeny Inference Package (PHYLIP, version 3.695) by Felsenstein (2008).

### Molecular phylogenetic analysis

We compared the partial sequence of the mitochondrial 16S rRNA gene of the samples from the different localities in Argentina to assess the number of species present in the country and their phylogenetic relationships (Table S3). The 16S barcoding gene is widely used, it amplifies with great success for most amphibians, there is a lot of available information already, and it is generally informative for tree-tips relationships, that is, among closely related species (Vences et al., 2005; Zhang et al., 2013). DNA was extracted using Qiagen DNeasy Blood and Tissue Kit (Qiagen, Hilden, Germany) following the manufacturer’s protocol. Polymerase chain reaction (PCR) was used to amplify fragments of approximately 560 bp of 16S mitochondrial rRNA using the standard primers 16SAL (5′-CGCCTGTTTACTAAAAACAT-3′), and 16SBH (5′-CCGGTCTGAACTCAGATCACGT-3′). Amplification followed the standard PCR conditions (Palumbi, 1996) with the following thermal cycle profile: 120 s at 94 °C, followed by 33 cycles of 94 °C for 30 s, 49 °C (12S)/53 °C (16S) for 30 s, and extension at 65 °C for 60 s. All amplified PCR products were verified using electrophoresis on a 1.4% agarose gel stained with ethidium bromide. PCR products were purified using Highpure PCR Product Purification Kit (Roche Diagnostics, Risch-Rotkreuz, Switzerland). Sequencing of both strands was performed with the DYEnamic ET Terminator Cycle Sequencing Premixkit (GE Healthcare, Munich, Germany) for sequencing reactions run on a MegaBACE 1000 automated sequencer (GE Healthcare, Munich, Germany). Chromas lite 2.1.1 software (Technelysium Pty Ltd, South Brisbane, Australia; http://www.technelysium.com.au) was used to check and read the chromatograms of the sequences. The obtained sequences were compared with those in GenBank using a standard nucleotide-nucleotide BLAST search. Homologous sequences of *Odontophynus* as well as from species of the closely related genera *Macrogenioglottus* and *Proceratophrys* were downloaded from GenBank and incorporated in an alignment. Sequences of *Pleurodema somuncurensis* (Leptodactylidae), *Rhinella marina* (Bufonidae) and *Ceratophrys cornuta* (Ceratophrydae) were used as outgroups (Table S3) to represent related anuran families (Feng et al., 2017). The sequences were aligned using the MUSCLE algorithm (Edgar, 2004) implemented in MEGA 7 (Kumar, Stecher & Tamura, 2016). The final alignment consisted of 552 bp. Pairwise distances were calculated in MEGA7. Distances >1% were considered indicative for differentiation at species level (Sáez et al., 2014).

The general time-reversible model with proportion of invariable sites and gamma-distributed rate variation among sites (GTR + I + G) was chosen as the best-fitting model of sequence evolution on the basis of the Akaike information criterion as implemented in jModelTest 2 (Darriba et al., 2012) and was applied in maximum likelihood (ML) and Bayesian inference (BI) analyses. ML was performed in MEGA 7 with heuristic searches with stepwise addition and TBR branch-swapping algorithm, generating 1,000 bootstrap replicates. BI was performed using MrBayes 3.2.5 (Ronquist et al., 2012). Two independent Metropolis-coupled Monte Carlo Markov Chain (Larget & Simon, 1999) analyses were run for 10 Million generations, each with one hot and three cold chains and the temperature set at 0.2. Trees were sampled every 5,000 generations. The first 500 samples of each run were discarded as burn-in, and the remaining trees from both runs were used to calculate a consensus tree and Bayesian posterior probabilities.

## Results

### Morphological variation

**Figure 1:**
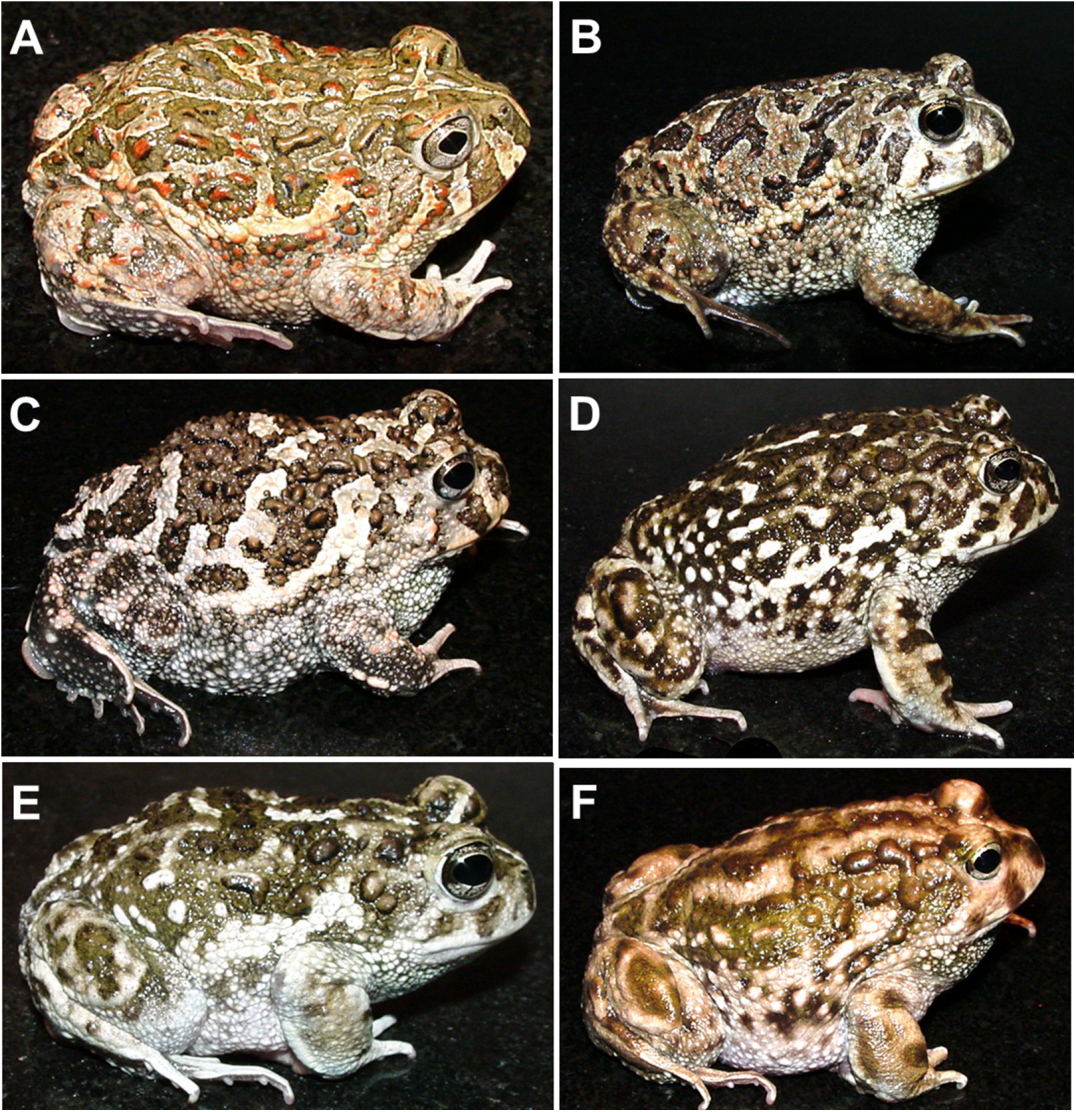
The nominal *Odontophrynus* taxa of Argentina. (A) *O. americanus*, (B) *O. cordobae*, (C) *O. lavillai*, (D) *O. occidentalis*, (E) *O. achalensis*, (F) *O. barrioi*. Dorsolateral view. Photographs by A. L. Martino.

**Figure 2:**
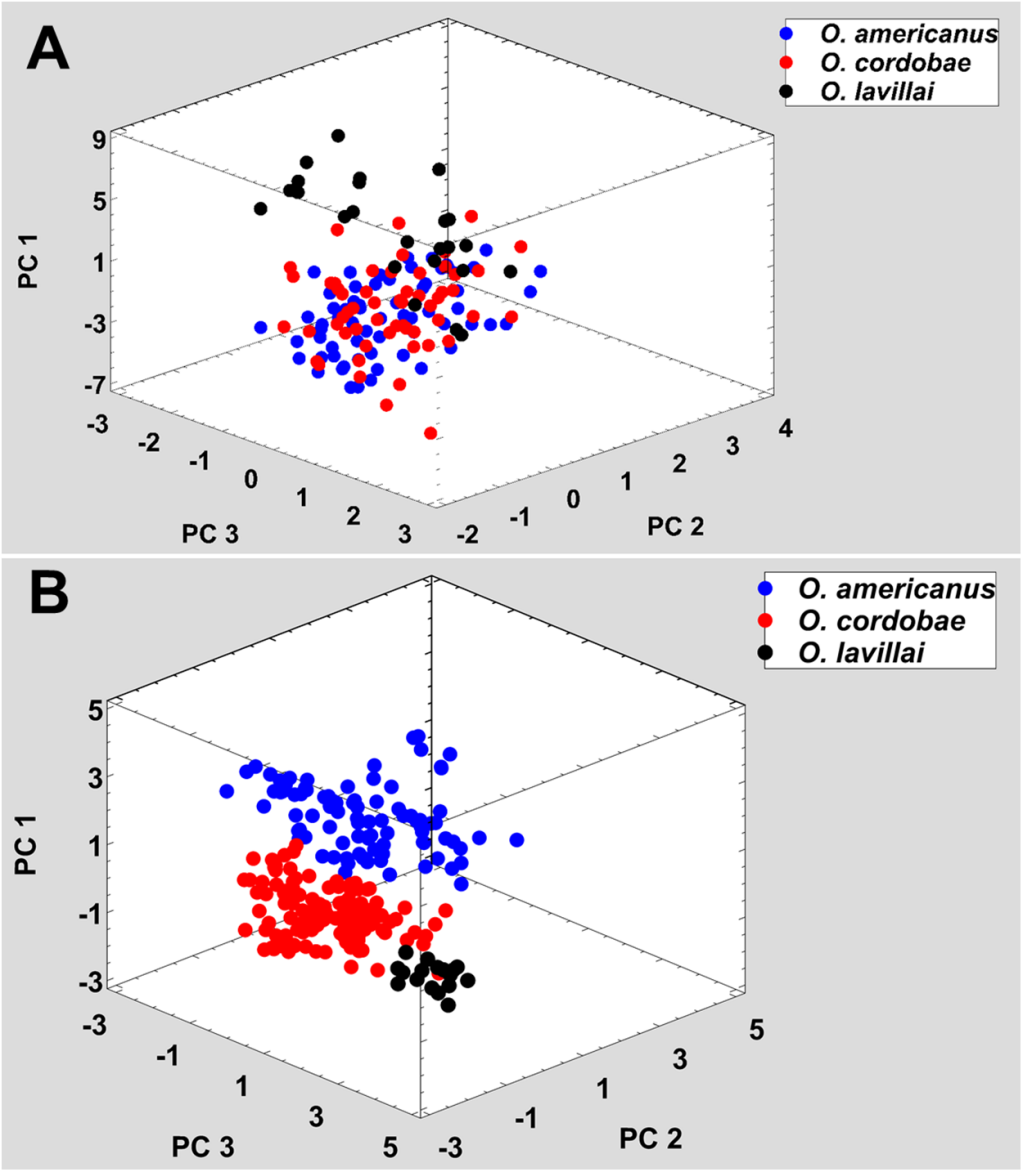
Phenotypic variation among the three nominal taxa included in the *Odontophrynus americanus* group. (A) Morphometric variation, (B) advertisement call variation. Each data point represents one individual.

**Figure 3:**
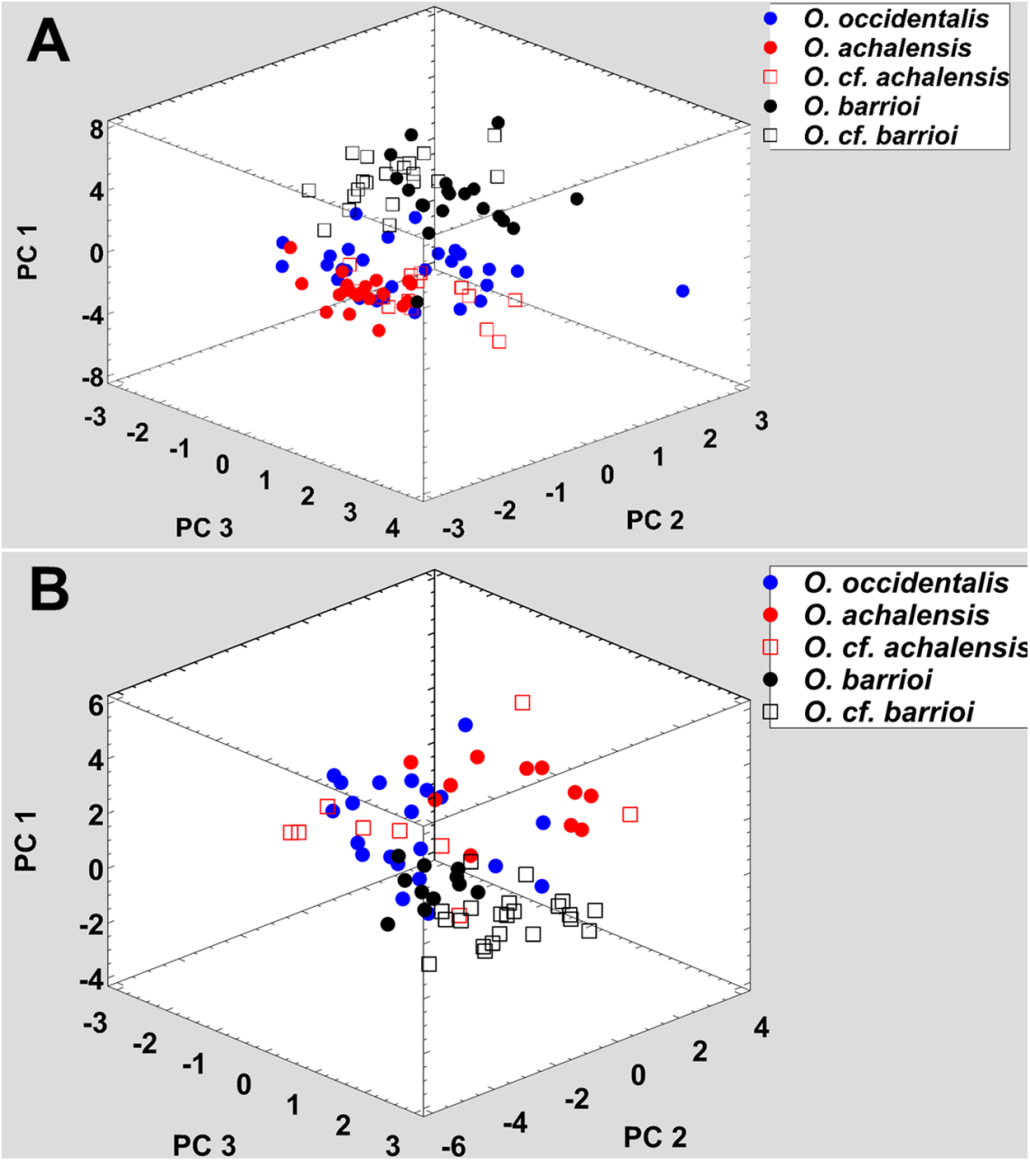
Phenotypic variation among the five nominal taxa included in the *Odontophrynus occidentalis* group. (A) Morphometric variation, (B) advertisement call variation. Each data point represents one individual.

All nominal taxa of *Odontophrynus* resemble each other considerably in coloration and external morphology reflecting their semi fossorial mode of living (Fig. 1). Quantitative morphometric analyses demonstrated a significant morphological variation among some taxa. The three principal component representing the axes of morphospace explained 77.2% of total variance in the *O. americanus* group and 80.1% in the *O. occidentalis* group, respectively (Table 1). The morphospace of the *O. americanus* group was partitioned between *O. lavillai* on one side and the indistinguishable pair *O. americanus/O. cordobae* on the other side (Fig. 2). The discriminant analysis confirmed a significant separation of *O. lavillai* with 85.7% of individuals correctly assigned to this species (Table 2). The taxa included in the *O. occidentalis* group showed a low morphometric differentiation with a wide overlap among *O. occidentalis, O. achalensis* and *O.* cf. *achalensis* (Fig. 3; Table 2). Classification success of discriminant function varied between 70% and 80% (Table 2). A significant proportion of morphometric variability among individuals assigned to the *O. occidentalis* group was caused by a clinal variation along altitudinal and latitudinal gradients. Size-related variation (PC1) was significantly correlated with altitude and latitude (Multiple regression model, *R*^2^ = 32.1%, *F*_2,102_ = 24.03, *P* < 0.00001), that is, size of individuals increased with elevation and from south to north. PC2 (position of nares and eyes) was significantly correlated with latitude (Multiple regression model, *R*^2^ = 16.6%, *F*_1,103_ = 20.52, *P* < 0.00001), PC3 (HL) with altitude (Multiple regression model, *R*^2^ = 10.0%, *F*_1,103_ = 11.42, *P* = 0.001).

**Table 2:** Discriminant functions based on a subset of standardized morphometric variables (Procedure: backward selection).

Discriminant function	Eigenwert	Percentage	Canonical correlation	Wilks lambda	Chi-squared	Degrees of freedom	*P*-value
*O. americanus*-group							
1	1.91	95.7	0.810	0.316	168.7	8	<0.00001
2	0.09	4.3	0.283	0.920	12.2	3	0.0067
*O. occidentalis*-group							
1	3.81	81.6	0.890	0.100	228.1	20	<0.00001
2	0.44	9.5	0.554	0.480	72.6	12	<0.00001
3	0.35	7.5	0.511	0.693	36.3	6	<0.0001
4	0.07	1.4	0.249	0.938	6.3	2	0.0420

	Standardized discriminant functions					
	*O. americanus*-group		*O. occidentalis*-group			
Variables	1	2	1	2	3	4
Maximum head width			0.130	0.682	−0.953	−0.311
Snout-eye distance			−0.141	0.895	0.641	0.186
Tibia length	0.692	−1.11	0.454	−1.028	−0.441	0.427
Length of 3rd finger	0.344	0.436	0.434	0.274	0.604	0.590
Length of 4th toe			0.386	−0.107	0.420	−0.938
Eye-narinal distance	0.370	0.333				
Rostronarinal distance	−0.268	1.051				

	Predicted species				
*Actual species*	*O. americanus*	*O. cordobae*	*O. lavillai*		
*O. americanus* (*n* = 66)	63.6% (*n* = 42)	36.4% (*n* = 24)	–		
*O. cordobae* (*n* = 57)	38.6% (*n* = 22)	57.9% (*n* = 33)	3.5% (*n* = 2)		
*O. lavillai* (*n* = 28)	–	14.3% (*n* = 4)	85.7% (*n* = 24)		

	Predicted species				
*Actual species*	*O. occidentalis*	*O. achalensis*	*O.* cf. *achalensis*	*O. barrioi*	*O*. cf. *barrioi*
*O. occidentalis* (*n* = 29)	75.9% (*n* = 22)	3.5% (*n* = 1)	10.3% (*n* = 3)	6.9% (*n* = 2)	3.5% (*n* = 1)
*O. achalensis* (*n* = 20)	–	80.0% (*n* = 16)	20.0% (*n* = 4)	–	–
*O.* cf. *achalensis* (*n* = 15)	6.6% (*n* = 2)	26.7% (*n* = 4)	77.3% (*n* = 11)	–	–
*O. barrioi* (*n* = 20)	–	–	5.0% (*n* = 1)	70.0% (*n* = 14)	25.0% (*n* = 5)
*O.* cf. *barrioi* (*n* = 21)	9.5% (*n* = 2)	–	–	19.1% (*n* = 4)	71.4% (*n* = 15)

**Note:**

Analyses were run separately on the two phenetic *Odontophrynus* groups. The subset include the smallest combination of measured variables that maximizes classification success. For details see text.

### Advertisement call variation

**Figure 4:**
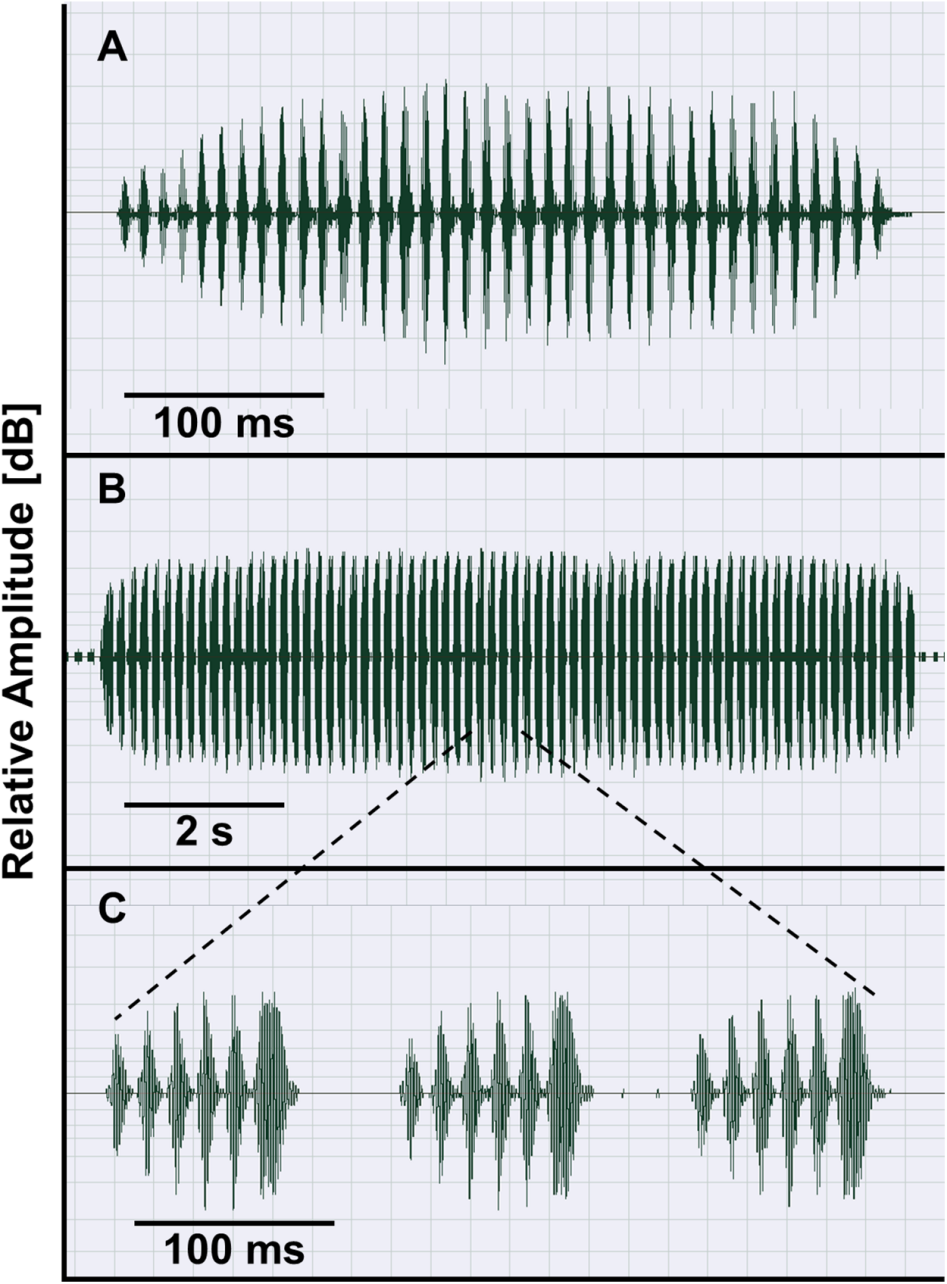
Advertisement calls of *O. americanus* (A) and *O. occidentalis* (B, C) as representatives of the two phenetic groups of *Odontophrynus* in Argentina. Oscillograms show calls recorded at 19.5 °C water temperature (A) and at 17.5 °C water temperature (B). Three pulse groups of the complex advertisement call of *O. occidentalis* (B) are presented in (C).

The taxa of the *O. americanus* group emit simple and short pulsed advertisement calls, whereas those of the *O. occidentalis* group produce long and complex advertisement calls consisting of a variable number of short pulse groups (Fig. 4). Quantitative analyses of the advertisement calls based on six temperature-adjusted variables in the *O. americanus* group showed a significant variation among the three taxa. Three PCs explained 85.3% of total variance represented the axes of sound space (Table 1). The sound space was partitioned into three discrete groups representing *O. americanus, O. cordobae* and *O. lavillai* individuals, respectively (Fig. 2). The discriminant analysis assigned all but five calls correctly to the corresponding taxon (Table 3).

**Table 3:** Discriminant functions based on based on a subset of standardized advertisement call variables (Procedure: backward selection).

Discriminant function	Eigenwert	Percentage	Canonical correlation	Wilks lambda	Chi-squared	Degrees of freedom	*P*-value
*O. americanus*-group							
1	6.67	84.1	0.933	0.058	633.5	10	<0.00001
2	1.26	25.9	0.747	0.443	181.1	4	<0.00001
*O. occidentalis*-group							
1	12.25	90.3	0.962	0.028	245.5	24	<0.00001
2	0.86	6.3	0.679	0.368	68.5	15	<0.00001
3	0.44	3.2	0.552	0.683	26.2	8	0.0010
4	0.02	0.2	0.137	0.981	1.30	3	0.7296

	Standardized discriminant functions					
	*O. americanus*-group		*O. occidentalis*-group			
Variables	1	2	1	2	3	4
Call duration	0.877	−0.429				
Pulse group duration			−0.636	−2.144	−0.107	−1.092
Interpulse group intervall			−0.888	−0.187	−0.200	0.562
Pulses per pulse group	−0.423	0.202	−0.503	1.450	−0.419	0.442
Pulse duration	0.275	−0.406	1.205	0.744	0.353	1.315
Interpulse group intervall			1.969	1.358	1.089	0.745
Pulse rate	−0.713	−0.465				
Dominant frequency	0.339	0.672	0.797	−0.157	−0.579	0.013

	Predicted species				
*Actual species*	*O. americanus*	*O. cordobae*	*O. lavillai*		
*O. americanus* (*n* = 91)	98.9% (*n* = 90)	–	1.1% (*n* = 1)		
*O. cordobae* (*n* = 119)	0.8% (*n* = 1)	96.6% (*n* = 115)	2.5% (*n* = 3)		
*O. lavillai* (*n* = 17)	–	–	100% (*n* = 17)		

	Predicted species				
*Actual species*	*O. occidentalis*	*O. achalensis*	*O.* cf. *achalensis*	*O. barrioi*	*O*. cf. *barrioi*
*O. occidentalis* (*n* = 21)	52.4% (*n* = 11)	23.8% (*n* = 5)	23.8% (*n* = 5)	–	–
*O. achalensis* (*n* = 11)	–	90.9% (*n* = 10)	9.9% (*n* = 1)	–	–
*O.* cf. *achalensis* (*n* = 10)	40.0% (*n* = 4)	20.0% (*n* = 2)	40.0% (*n* = 4)	–	–
*O. barrioi* (*n* = 11)	–	–	–	100% (*n* = 11)	–
*O.* cf. *barrioi* (*n* = 22)	–	–	–	4.6% (*n* = 1)	95.4% (*n* = 21)

**Note:**

Analyses were run separately on the two phenetic *Odontophrynus* groups. The subset include the smallest combination of measured variables that maximizes classification success. For details see text.

Analogous to morphometric variation, sound space partitioning was low among the taxa of the *O. occidentalis* group, with *O. occidentalis, O. achalensis* and *O.* cf. *achalensis* being indistinguishable among each other (Fig. 3; Table 3). The sound space occupied by *O. barrioi* and *O.* cf. *barrioi* differed from the continuum formed by the other taxa, but showed a slight overlap between each other. Still, the correct classification rates for *O. barrioi* and *O.* cf. *barrioi* were 100% and 95.4%, respectively (Table 3). Temperature-adjusted advertisement call variation was also influenced by geographical clines. PC1 (size-related dominant frequency) and PC2 (call duration) were significantly correlated with latitude (Multiple regression models: *R*^2^ = 9.8%, *F*_1,74_ = 7.94, *P* = 0.0062, and *R*^2^ = 14.1%, *F*_1,74_ = 11.97, *P* = 0.0009, respectively), and PC3 (pulse group duration) with altitude (*R*^2^ = 9.1%, *F*_1,74_ = 7.31, *P* = 0.0085).

### Genetic distances: allozymes and barcoding

A total of 14 putative loci were scored in the nominal taxa (Table S2). Two loci (mAAT, mMDH) were monomorphic in all taxa. The overall degree of allele fixation was high and varied between five loci in *O. americanus* and 11 in *O. lavillai*. Five taxa possessed one private allele each: *O. americanus* cIDH a, *O. lavillai* cAAT a, *O. achalensis* LDH d, *O.* cf. *achalensis* GPI d and *O. barrioi* cMDH a. The pairwise Nei distances among the taxa were clearly below species level in four taxon pairs (Table 4): 0.0220 in *O. americanus/O. cordobae,* 0.0232 in *O. achalensis/O. occidentalis,* 0.0292 in *O.* cf. *achalensis/O. occidentalis,* and 0.0351 in *O. achalensis/O.* cf. *achalensis*.

**Table 4:** Nei’s genetic distances among six nominal *Odontophrynus* taxa and and two achalensis- and barrioi-like populations.

Taxon	*O. cordobae*	*O. lavillai*	*O. occidentalis*	*O. achalensis*	*O.* cf. *achalensis*	*O. barrioi*	*O.* cf. *barrioi*
*O. americanus*	0.0220	0.1853	0.1821	0.1942	0.2452	0.4196	0.5471
*O. cordobae*		0.2224	0.2084	0.2146	0.2707	0.4160	0.5943
*O. lavillai*			0.2781	0.4126	0.4982	0.6705	0.5608
*O. occidentalis*				0.0232	0.0292	0.1846	0.2604
*O. achalensis*					0.0351	0.1660	0.3422
*O.* cf. *achalensis*						0.1772	0.3406
*O. barrioi*							0.2186

**Note:**

Distances were calculated from the allele frequencies listed in S2.

**Figure 5:**
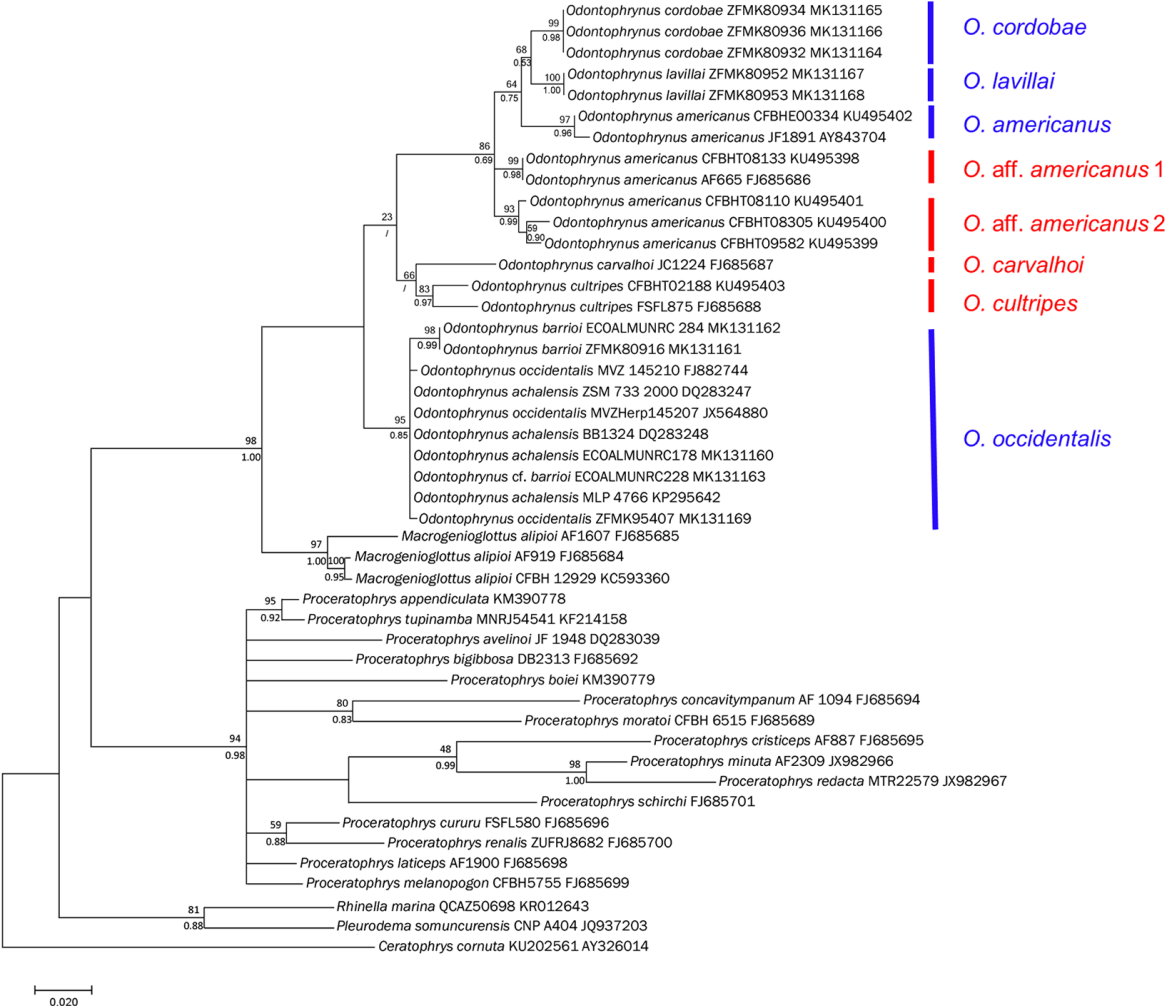
Bayesian phylogram on Odontophrynidae inferred from mitochondrial nucleotide sequence data of 16S rRNA. Numbers above branches are non-parametric bootstrap support values from MP and ML, respectively, numbers below branches are Bayesian posterior probabilities. In two cases with low bootstrap support, the nods were not supported by Bayesian approach. Blue bars indicate the four genetically delimited species occurring in Argentina, red bar the species occurring in Brazil. Please note that *O.* aff. *americanus* 1 and 2 are undescribed taxa which differ from the topotypical *O. americanus* from Argentina and southern Brazil.

The 25 samples from eight nominal *Odontophrynus* species differed from each other in the 16S sequences by uncorrected pairwise distances of 0.0–7.3% (Table 5). The divergence between samples of *O. achalensis*, *O. barrioi*, *O.* cf. *barrioi* and *O. occidentalis* were minimal (0.0–0.9%) and we regard them as belonging to a single species. The distances among the three nominal species *O. americanus*, *O. cordobae* and *O. lavillai* collected in Argentina were at species level (1.8–2.7%). Interestingly, the specimens referred to as *O. americanus* from Brazil (Amaro, Pavan & Rodrigues, 2009; Lyra, Haddad & De Azeredo-Espin, 2017) resolved into three groups differing at species level among each other (2.4–4.0%). The topotypic *O. americanus* from Argentina was conspecific with the Rio Grande specimen (southern Brazil; *P*-distance = 0.4 %). Genetic differentiation among the specimens collected in Argentina delimits four *Odontophrynus* species (Fig. 5).

**Table 5:** Uncorrected *P*-distances (%) among the nominal *Odontophrynus* taxa of Argentina, *O. americanus* samples from Brazil and *M. alipioi, P. bigibossa* and *C. cornuta* (outgroups).

Taxon	*O. americanus* (Minas Gerais, Brazil)	*O. americanus* (Sao Paulo, Santa Catarina, Brazil)	*O. cordobae*	*O. lavillai*	*O. occidentalis*	*O. achalensis*	*O. barrioi*	*O.* cf. *barrioi*	*M. alipioi*	*P. bigibossa*	*C. cornuta*
*O. americanus* (Buenos Aires, Argentina, Rio Grande, Brazil)	2.4–3.5	2.4–4.0	2.1	2.5–2.7	4.6–5.2	4.6–5.0	5.2–5.6	5.0	5.9–6.2	8.7–9.3	10.8
*O. americanus* (Minas Gerais, Brazil)		2.4–3.0	1.6–2.4	2.4–3.2	4.2–6.5	5.0–6.2	4.7–7.0	4.2–6.2	6.2–6.4	9.6	11.0
*O. americanus* (Sao Paulo, Santa Catarina, Brazil)			3.5–4.0	3.5–3.8	5.5–6.8	5.5–6.5	6.3–7.3	5.5–6.5	6.4–7.3	11.1–11.7	11.9–12.1
*O. cordobae*				1.8	4.6–4.7	4.6	5.1	4.6	5.7–6.0	8.8	10.6
*O. lavillai*					4.6–4.7	4.6	5.1	4.6	6.8–6.9	9.2	11.2
*O. occidentalis*						0.0–0.2	0.7–0.9	0.2	3.8–5.1	8.8–9.0	9.9–10.1
*O. achalensis*							0.7	0.0	3.8–4.9	8.8	9.9
*O. barrioi*								0.7	3.8–5.3	8.6	9.7
*O.* cf. *barrioi*									3.8–4.9	8.8	9.9
*M. alipioi*										8.8–9.0	9.5–10.6
*P. bigibossa*											11.0

**Note:**

Distances were calculated using the partial sequences of the mitochondrial 16S rRNA gene listed in S3.

### Phylogenetic relationships among the Odontophrynidae

The topologies derived from the two phylogenetic analysis methods were largely congruent. We show the BI phylogeny with bootstrap values from ML and posterior probabilities from BI (Fig. 5). In agreement with earlier phylogenies (Amaro, Pavan & Rodrigues, 2009; Pyron & Wiens, 2011; Dias et al., 2013; Feng et al., 2017) our data support the monophyly of the three genera within Odontophrynidae and the sister group relationship between *Macrogenioglottus* and all *Odontophrynus* taxa. The *Proceratophrys* clade resolved as the sister group to the clade formed by the other two genera. The samples of *Odontophrynus* resolved into two major clades with strong node support. The first one contained the samples of *O. occidentalis* as well as those of *O. achalensis* and *O. barrioi*. The relationships within this clade remained largely unresolved and the three nominal taxa did not separate into distinct phylogenetic lineages. The second clade contained the remaining species divided into two subclades, one consisting of *O. carvalhoi* and *O. cultripes*, the other one containing *O. americanus*, *O. cordobae* and *O. lavillai*. The samples of *O. americanus* did not form a monophyletic clade since the topotypic Argentinian sample and the specimen from southern Brazil were more closely related to *O. cordobae* and *O. lavillai* than to the other Brazilian samples referred to as *O. americanus*.

### Synonymy and updated diagnosis of *O. occidentalis* (Berg, 1896)

Since we did not detect significant genetic differentiation among *O. achalensis, O. barrioi* and *O. occidentalis,* we consider these three nominal taxa as a single species. Applying the Principle of Priority of the International Code of Zoological Nomenclature (International Commission on Zoological Nomenclature, ICZN, 1999), the oldest available name is *O. occidentalis*.

**Synonymy.** Besides the names *C. occidentalis* Berg, 1896 and *O. occidentalis* Müller, 1934, we place the names *O. barrioi* Cei, Ruiz, and Beçak, 1982, and *O. achalensis* Di Tada et al., 1984 into the synonymy of *O. occidentalis* (Berg, 1896).

**Holotype.** Male (MACN 380), housed in the Museo Argentino de Ciencias Naturales “Bernardino Rivadavia”, Buenos Aires, Argentina (Rosset et al., 2007). Type locality: “Arroyo Agrio (Neuquén)”, Argentina.

**Molecular diagnosis.** The partial mitochondrial 16S RNA gene sequences of 10 specimens collected throughout the geographical range (Table S3) are unique within the genus *Odontophrynus,* show a low within-taxon range of variation (0.0–0.9% uncorrected *P*-distances) and differ significantly from the corresponding sequences of *O. americanus, O. cordobae* and *O. lavillai* (4.6–5.2% uncorrected *P*-distances; Table 5) originating from Argentina.

**Karyological diagnosis.** The karyotype of *O. occidentalis* is diploid with 2*n* = 22 chromosomes as in *O. cordobae* and in *O. lavillai*, distinguishing these species from the tetraploid *O. americanus* (Barrio & Pistol de Rubel, 1972; Cei, Ruiz & Beçak, 1982; Ruiz, Cei & Beçak, 1982; Salas, Steinlein & Schmid, 2000; Rosset et al., 2006; Salas & Martino, 2007). Secondary constrictions are present on pair 4 in some *O. americanus*, all *O. cordobae* and *O. lavillai* and on pair 8 in *O. cordobae* and on pair 11 in *O. americanus* and *O. occidentalis* (Barrio & Pistol de Rubel, 1972; Rosset et al., 2006; Salas & Martino, 2007). The intercalary C-band on chromosome 9 is characteristic for all studied *O. occidentalis* populations, that is, present in the achalensis, barrioi and occidentalis ecotypes, but absent in the other *Odontophrynus* spp. (Ruiz, Cei & Beçak, 1982). C-banding patterns shows some variability among populations, with additional bands on chromosome 1 (barrioi), 2 and 3 (barrioi, occidentalis), and 4 (achalensis) (Ruiz, Cei & Beçak, 1982; Salas, Steinlein & Schmid, 2000). *Odontophrynus occidentalis* is diagnosed karyologically by the combination of 2*n* = 22 chromosomes, secondary constrictions on chromosome pair 11 and an intercalary C-band on chromosome 9.

**Morphological diagnosis of adults.** *Odontophrynus occidentalis* are short-legged and stout as all described *Odontophrynus* toads. Enlarged to rounded paratoid glands are present. Sexual size dimorphism absent. Individuals of lowland populations on average smaller than those of highland populations. In life, the dorsal color is brownish with brown spots and faint lateral and dorsal yellowish longitudinal bands, expression of light vertebral stripe variable from absent to short at the head or urostyle (Fig. 1). Ventral background color is whitish with scattered white granuli.

Further qualitative features are in wide agreement with the original descriptions in Berg (1896), Cei, Ruiz & Beçak (1982), Di Tada et al. (1984), Cei (1985) and Rosset et al. (2007). (1) Nostrils nearer to the snout than to the eyes, in lateral profile; (2) lateral fringes poorly or not developed on fingers; (3) metatarsal tubercle shovel-like, moderately developed; (4) short fore and hind limbs; (5) ED 1.7 times greater than eye-nostril distance; (6) interorbital distance longer than the upper eyelid width; (7) tibia slightly shorter than femur; (8) HL approximately one third of the SVL; (9) keratinous spines on the dorsum present mainly in highland populations. Among- and within-population variation exist in shape, size and number of the dorsal glands and in the presence and number of keratinous spines on the dorsum (Di Tada et al., 1984; Rosset et al., 2007).

Crespo & Cei (1983) proposed several morphometric ratios (tibiofibula length/fourth toe length, sacrum width/total length, sacrum width/vertebral column length, skull length/total length) to distinguish barrioi from occidentalis ecotypes. Rosset et al. (2007) rejected this proposal. Quantitative features (median, range in [mm]) of 92 males and 13 females are calculated irrespective of altitude and latitude at collection locality. (1) SVL: 54.1 (36.3–71.2, males) vs. 51.0 (42.0–71.2, females); (2) HW: 24.5 (15.4–29.6) vs. 24.4 (19.2–31.4); (3) HL: 18.3 (11.1–24.9) vs. 17.8 (13.1–23.4); (4) SED: 8.6 (5.1–10.9) vs. 8.1 (5.6–10.2); (5) IND: 4.0 (3.1–5.5) vs. 3.8 (2.7–5.6); (6) IOD: 3.6 (2.6–4.7) vs. 3.9 (2.8–5.1); (7) END: 4.5 (3.1–6.5) vs. 4.4 (2.8–6.1); (8) RND: 5.4 (3.3–6.7) vs. 5.0 (3.0–6.3); (9) ED: 7.5 (5.6–10.4) vs. 7.4 (5.5–9.8); (10) HL: 24.4 (15.9–33.4) vs. 24.4 (17.5–34.5); (11) F3L: 7.6 (5.9–11.6) vs. 8.1 (5.9–11.5); (12) FL: 22.3 (15.5–32.0) vs. 20.4 (15.1–29.8); (13) TL: 20.0 (13.4–26.8) vs. 19.8 (14.5–25.4); (14) FOL: 33.0 (22.4–45.1) vs. 31.5 (24.1–46.6); (15) T4L: 11.1 (6.9–16.7) vs. 10.6 (7.9–18.4).

**Morphological diagnosis of tadpoles.** In the exotrophic, lentic and benthic *Odontophrynus* spp. tadpoles, the body is ovoid in dorsal and ventral views and slightly depressed in lateral view, the snout is circular in dorsal and ventral views with an anteroventral oral disc, the eyes and nostrils placed dorsally, and the spiracle sinistral, short, and with an opening posterodorsally directed (Do Nascimento et al., 2013). *Odontophrynus occidentalis* differs from the other species by being considerably larger at stages 36–38 (average: 71.7 mm; maximum: 75 mm (Cei, 1987). Do Nascimento et al. (2013) and González et al. (2014) state that external tadpole morphology is very similar in the achalensis, barrioi and occidentalis ecotypes, unlike Cei & Crespo (1982). Shared characters apart from the general *Odontophrynus* spp. features are a tail, that is, larger than half of the total length with robust musculature and 2(2)/3(1) as labial tooth row formula. Among the distinguishing characters mentioned by Cei et al. (1982), Cei & Crespo (1982) and Cei (1987) for the barrioi ecotype, only the diverging form of the tip of tail (rounded contrary to acuminate in occidentalis and achalensis) has been confirmed by González et al. (2014). The significance of this difference remains unclear because phenotypic plasticity of tail morphology in response to syntopic predators is well-documented (Van Buskirk, 2009).

**Bioacoustic diagnosis.** *Odontophrynus occidentalis* is unique among all currently known *Odontophrynus* species by giving series of complex advertisement calls consisting of a variable number of pulse groups (Figs. 4B and 4C). Rosset et al. (2007) state that advertisement call features in the ecotypes achalensis, barrioi and occidentalis are very similar. The bioacoustic distinction of achalensis from occidentalis (Salas & Di Tada, 1994) and of barrioi from the other ecotypes (Rosset et al., 2007) is based mainly on differences in the dominant frequency (related to body size), in call duration (sensitive to environmental temperature), and in intercall interval (sensitive to behavioral interactions such as chorusing; Schneider & Sinsch, 2007). In fact, temperature-adjusted dominant frequency Standardized Dominant Frequency (SDF) was significantly correlated with SVL of the caller demonstrating that ecotype distinction reflects mainly size variation among populations (Linear regression model: SDF = 2.405–0.065*SVL, *R*^2^ = 0.623; *F*_1,74_ = 120.6, *P* < 0.0001; Fig. S1). SVL- and temperature-adjusted call duration did not differ significantly among the ecotypes (ANOVA, *F*_4,74_ = 2.14, *P* = 0.084).

The following features (median, range, *n* = 229) of *O. occidentalis* advertisement calls are calculated irrespective of the size of the calling individual and of ambient temperature, altitude and latitude at recording locality. (1) Call duration 2,619 ms (767–64,303 ms); (2) pulse groups per call 18 (5–348); (3) pulse group duration 98 ms (60–171 ms); (4) pulses per pulse group 7 (5–11); (5) interpulse group interval 57 ms (38–97 ms); (6) pulse duration eight ms (4–13 ms); (7) interpulse interval six ms (2–16 ms); (8) pulse rate: 48.3 pulses/s (24.9–65.3 pulses/s); and (9) dominant frequency 754 Hz (553–955 Hz). Features of the encounter call are given in Rosset et al. (2007).

**Geographic and habitat range.** Populations are present in a latitudinal range from 41°S in the Rio Negro Province to 27.5°S in the north of the Catamarca Province (Ruiz, Cei & Beçak, 1982; Rosset et al., 2007). The corresponding altitudinal range is from 690 m above sea level to 1,854 m in the Sierra Pie de Palo, 1,965 m in the Sierra de San Luis, 2,149 m in the Sierra de Achala, and 2,200 m in the Sierra de Famatina (Table S1). Terrestrial habitats include pastureland, montane grassland, shrubs, forests and rock outcrops. Aquatic habitats used for reproduction and larval development are exclusively creeks and slowly running rock pool sections of permanent streams.

## Discussion

Lines of evidence obtained from phenotypic and genotypic character complexes in *Odontophrynus* toads exemplify the common dilemma of taxonomy—which level of character differentiation requires taxonomic consequences? Our case study demonstrates that phenotypic plasticity may result in an overestimation of species number (*O. occidentalis* group), whereas molecular data may reveal unexpected cryptic diversity in morphologically uniform populations (referred to as *O. americanus*). The following discussion of the three hypotheses basic to our investigation will present a revised view on the actual *Odontophrynus* species richness and propose a model of the phylogenetic relationships within the genus *Odontophrynus*.

### Hypothesis 1: Phenotypic plasticity within and among *Odontophrynus* taxa is associated with corresponding genetic differentiation

Applying the consensus protocol for integrative taxonomy (Padial et al., 2010) we evaluate the support for the genetically delimited species *O. americanus, O. cordobae, O. lavillai* and *O. occidentalis* by the among-taxon variation of allozyme, morphology and bioacoustic character complexes. Congruence of genetic and phenotypic differentiation clearly delimits *O. lavillai* from the congeneric taxa in Argentina. Independent support for its distinction from the only sympatric *Odontophrynus* species stems from karyological features, that is, *O. lavillai* is diploid whereas topotypic *O. americanus* is tetraploid (Barrio & Pistol de Rubel, 1972; Cei, 1985; Rosset & Baldo, 2014). Natural hybrids between these species have not been detected.

The significant differentiation of 16S RNA gene sequence distinguishes between the tetraploid *O. americanus* and the diploid *O. cordobae* as does the advertisement call structure (Martino & Sinsch, 2002; this study). Congruent allozyme and morphometric differentiation seem to be absent (Barrio & Pistol de Rubel, 1972; this study). However, adjusting size by age reveals that the two species differ significantly in their ontogenetic growth trajectories (Martino & Sinsch, 2002) supporting the molecular distinction. Premating isolation mechanisms allow the two species to maintain reproductive isolation in the narrow zone of sympatry, as indicated by a very low incidence of triploid hybrids at only two localities (Grenat et al., 2018). Thus, cumulating available evidence on character differentiation (except for the allozyme pattern) supports the species status of these two taxa.

To our surprise, the 16S RNA gene sequence of a topotypical tetraploid *O. americanus* from the Buenos Aires province, Argentina and that of five specimens of unknown ploidy referred to as *O. americanus* from different localities in Brazil (Amaro, Pavan & Rodrigues, 2009; Lyra, Haddad & De Azeredo-Espin, 2017) differed so profoundly that we consider them as a complex of three distinct species. One sample from Rio Grande, Brazil proved to be very similar to the topotypical tetraploid *O. americanus* indicating that this species extends over Uruguay to southern Brazil. A second group of two specimens originating from southern Minas Gerais is termed here as *O.* aff. *americanus* 1 and a distinct species from topotypical tetraploid *O. americanus.* Its distinction from or similarity with the diploid *O. juquinha* from northern Minas Gerais remains to be studied in the future. Finally, a third group of three specimens from Sao Paulo and Santa Catarina provinces, termed here as *O.* aff. *americanus* 2, is a distinct species from Minas Gerais taxon and true *O. americanus.* One of these specimens originated from Irai which is about 100 km distant from the “*O. americanus*”-populations reported from the province Misiones, Argentina (Rosset et al., 2006). It seems reasonable to assume that the geographical range of *O.* aff. *americanus* 2 extends to Argentina in the west, but further studies are needed to resolve the relationship to the Misiones populations.

In contrast, we did not detect any significant genetic differentiation among the specimens belonging to different nominal taxa of the *O. occidentalis* group despite a considerable phenotypic plasticity among and within populations. Broadly overlapping variation in all character complexes surveyed demonstrates that *O. achalensis* from the Sierra de Cordoba and associated populations from the Sierra de San Luis are phenotypically and genetically indistinguishable from *O. occidentalis*. Sympatry of the two forms in the Sierra de Cordoba suggests ongoing gene flux between lowland and highland phenotypes. The taxonomic conclusion is straightforward—*O. achalensis* does not deserve species status; it is simply the highland ecotype of the eastern and central part of the *O. occidentalis* range. Applying the Principle of Priority of the International Code of Zoological Nomenclature (International Commission on Zoological Nomenclature, ICZN, 1999), we consider *O. achalensis* a junior synonym of *O. occidentalis*.

The case of *O. barrioi* is more complicated because the absence of significant genetic differentiation from *O. occidentalis* contrasts with considerable morphometric, bioacoustic and allozyme differentiation. The *O. barrioi* populations vary morphometrically only by their greater size whereas shape variation is the same as in the *O. occidentalis/O. achalensis* continuum. Within-species altitudinal and latitudinal size variation is well known in anurans (Sinsch, Pelster & Ludwig, 2015), and SVL differences alone appear to be poor indicators of species distinction (Rakotoarison et al., 2012; Rojas et al., 2016). Advertisement call variation is mainly based on differences in dominant frequency, again an indicator of size of the calling individual (Fig. S1) and thus, of low taxonomic significance. The features of qualitative external morphology proposed by Rosset et al. (2007) to diagnose *O. achalensis, O. barrioi* and *O. occidentalis* represent the extremes of a continuum between lowland and highland ecotypes, and between eastern and northern variants of the same species. For the same reason González et al. (2014) failed to detect significant morphological differences among the tadpoles within the *O. occidentalis* group refuting the claim of Cei & Crespo (1982). Moreover, defensive behavior of adults is also indistinguishable (Borteiro et al., 2018). Thus, the phenotypic peculiarities of *O. barrioi* seem responses to local environmental conditions rather than indicating taxonomically relevant information. Applying the Principle of Priority of the International Code of Zoological Nomenclature (International Commission on Zoological Nomenclature, ICZN, 1999), we consider *O. barrioi* a junior synonym of *O. occidentalis*.

In conclusion, we verify hypothesis 1 for the species of the *O. americanus* group, but not for the nominal taxa of the *O. occidentalis* group. Conflicting evidence from phenotypic and genotypic variation in the taxa of the *O. occidentalis* group demonstrates that adaptation to altitude and geographic isolation from conspecific populations (allopatry) may result in phenotypes that were erroneously referred to as distinct species. Molecular evidence melts down the *O. occidentalis* group to single, polymorphic and highly adaptable species *O. occidentalis*.

### Hypothesis 2: The phenetic groups within *Odontophrynus* represent distinct phylogenetic lineages

Our phylogram is formed by three clades within *Odontophrynus* representing the morphologically defined *O. americanus*, *O. cultripes* and *O. occidentalis* groups (Fig. 5). The basal splitting of lineages separates *O. occidentalis*, the only species with complex advertisement call consisting of several pulse groups, from the two lineages with simple pulsed calls. The ancestral character state of advertisement call structure in Odontophrynidae is most likely a simple pulsed call, as also present in the sister group *Macrogenioglottus alipioi* (Abravaya & Jackson, 1978) and in most *Proceratophrys.* The species occurring in Argentina and Bolivia, the diploids *O. cordobae* and *O. lavillai*, and the topotypical tetraploids *O. americanus* are closely related, but the sister species relationship between *O. americanus* and *O. cordobae* is well resolved possibly indicating an autopolyploid origin of these tetraploids.

In conclusion, we verify hypothesis 2 with respect to the genetic base of the phenetic groups. Our reconstruction of phylogenetic relationships among these groups suggests that *O. occidentalis* evolved from the ancestral stock before the diversification of the *O. americanus* and *O. cultripes* group occurred.

### Hypothesis 3: The current Red List classification does not reflect the risks upon each species

**Figure 6:**
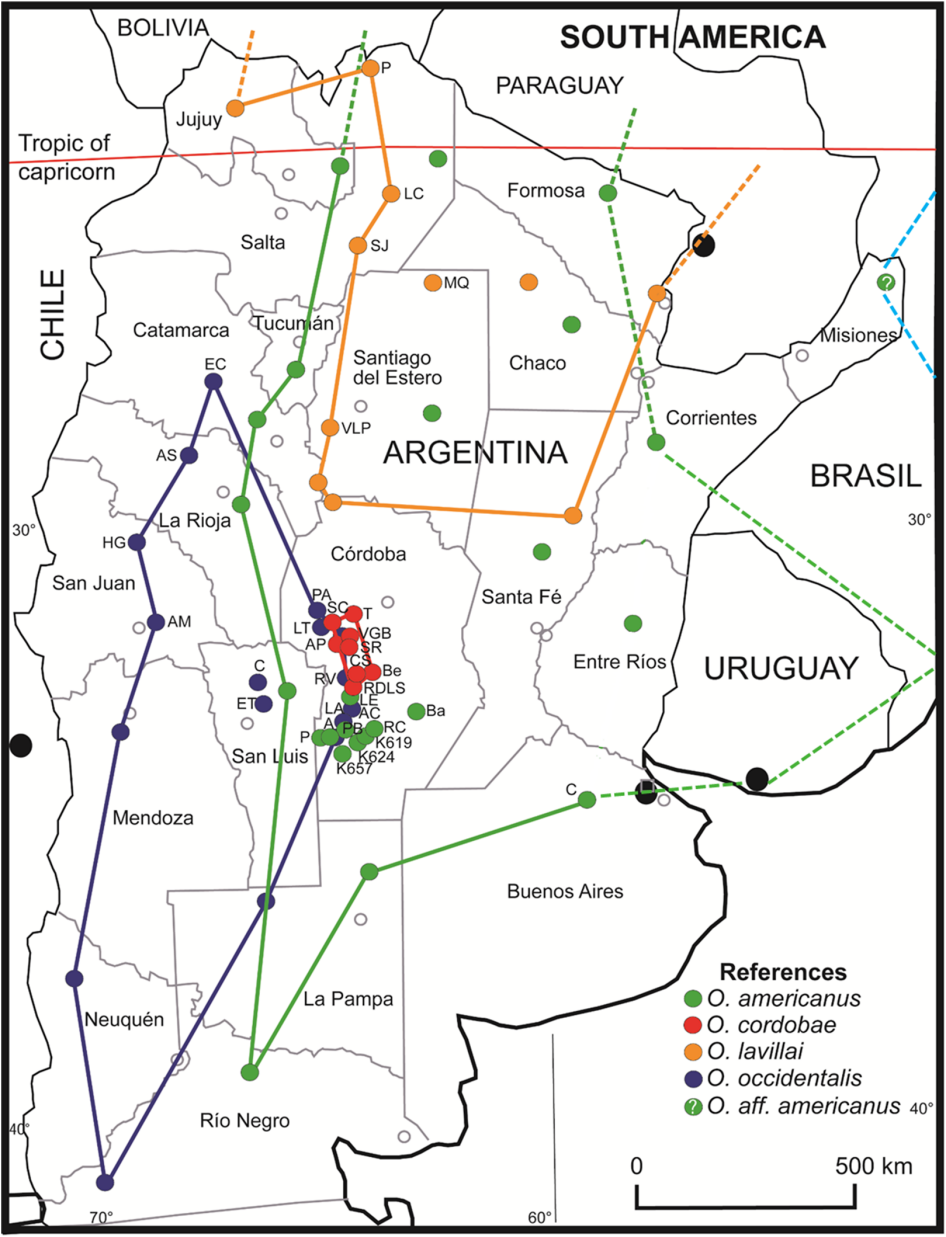
Geographic distribution of *Odontophrynus* species for eight provinces and 34 different localities sampled. *Odontophrynus americanus* (green label). Córdoba province (nine localities): A, Achiras; BA, Barreto; K619, Km 619, National Road #8; K624, Km 624, National Road #8; K657, Km 657, National Road #8; LE, La Escondida; P, Punilla; PB, Piedra Blanca; RC, Río Cuarto. Buenos Aires province (one locality): C, Chivilcoy. *O. cordobae* (red label). Córdoba province (eight localities): AP, Athos Pampa; Be, Berrotarán; CS, Cañada del Sauce; RDLS, Río de los Sauces; SC, San Clemente; SR, Santa Rosa; T, Tanti; VGB, Villa General Belgrano. Note that the range extends to Bolivia, Paraguay, Uruguay and southern Brazil (dashed lines). *O. lavillai* (orange label). Santiago del Estero province (two localities): MQ, Monte Quemado; VLP, Villa La Punta. Salta province (three localities): LC, Los Colorados; P, Pocitos; SJ, San Javier. Note that the range extends to Bolivia and Paraguay (dashed lines). *O. occidentalis* (blue label). Córdoba province (seven localities): A, Achiras; AP, Alpa Corral; LA, Las Albahacas; LT, Est. Los Tabaquillos; PA, Pampa de Achala; RV, Rodeo Viejo; VGB, Villa General Belgrano. San Luis province (two localities): C, Carolina; ET, El Trapiche. San Juan province (two localities): AM, Aguada del Molle, Sierra de Pié de Palo; HH, Huerta de Guachi. La Rioja (one locality): AS, Aguadita Springs. Catamarca province (one locality): RC, Río El Carrizal, Condor Huasi. *Odontophrynus* aff. *americanus* (pale blue label). Misiones province; probably identical with an undescribed *americanus*-like species from Brazil. Details on localities are given in Table S1.

The geographical distribution of *O. occidentalis* is larger than previously appreciated extending to north (barrioi ecotype) and to east (achalensis ecotype) (Fig. 6). *Odontophrynus occidentalis* is endemic to Argentina inhabiting many localities in eight provinces covering about 16% of the territory. This species is highly adaptable to a wide altitudinal range, and tolerant to local sympatry with *O. americanus* and *O. cordobae*. Thus, the Red List classification “Least Concern” seems justified, whereas the associated ecotypes “achalensis” and “barrioi” (“Vulnerable” and “Data Deficient”) do not deserve classifications apart. With respect to the species referred to as *O. americanus* our study suggests strongly that there is more than one species involved. The nominal species *O. americanus* is certainly widespread in Argentina (16 provinces and ca. 67% of the territory) and extends to Bolivia and Paraguay in the north, and to Uruguay and southern Brazil in the east. The status “Least Concern” seems appropriate. The exact range of this taxon in Brazil remains to be assessed using barcoding for species identification. Most probably, the easternmost locality in Misiones pertains rather to *O*. aff. *americanus* 2 of Brazil than to the nominal taxon of Argentina. If this assumption proves true, the still undescribed *Odontophrynus* taxon would constitute the fifth species of this genus in Argentina. *O. cordobae* has the smallest area of distribution of the four species, occurs exclusively in the central part of the Cordoba province, and thus, is endemic to Argentina (Fig. 6). Recent assessment of localities inhabited demonstrates that there is a viable network of probably connected populations (Grenat et al., 2018). Therefore, we propose the classification “Least Concern” as long as there is no further shrinkage of its geographical range. Finally, *O. lavillai* inhabits eight provinces of Argentina as does *O. occidentalis*, but its range extends further north to Bolivia and Paraguay (Rosset & Baldo, 2014). The classification “Least Concern” seems reasonable for this species as well. The Red List status of newly described species from Brazil and Uruguay and those of the *O. cultripes* group are outside the scope of this study.

In conclusion, we falsify hypothesis 3 with respect to genetically delimited *Odontophrynus* species of Argentina.

## Conclusions

Integrative taxonomy has proved to be the appropriate tool to cope with distinct levels of character differentiation in the morphologically highly conserved genus of *Odontophrynus* toads. Genotypic variation among the nominal taxa of the *O. occidentalis* group did not correspond to the phenotypic plasticity in response to altitudinal and latitudinal gradients found in the ecotypes “achalensis”, “barrioi” and “occidentalis”. Consequently, molecular evidence melts down the *O. occidentalis* group to a single, polymorphic species *O. occidentalis*. Whereas the number of species was grossly overestimated in this case, considerable genetic divergence between *O. americanus* originating from a topotypical population (Argentina) and specimens of unknown ploidy from Brazil indicates that specimens referred to as *O. americanus* in Argentina and in Brazil are distinct species except for the southernmost populations in Brazil. Phylogenetic relationships among *Odontophrynus* species suggest that *O. occidentalis* evolved from the ancestral stock before the diversification of the *O. americanus* and *O. cultripes* group occurred. Reliable taxonomic delimitation of *Odontophrynus* taxa allows for a precise assessment of the corresponding geographical ranges and for an informed basis of the Red List classification. The four species occurring in Argentina do not seem endangered currently, but the small geographic range of *O. cordobae* may require a future reassessment of the species’ status.

## Supplemental Information

10.7717/peerj.6480/supp-1 Supplemental Information 1 List of *Odontophrynus* taxa collected from 34 localities in Argentina. All specimens were examined for morphometric analyses. Tissue samples used for allozyme and barcoding sequences were obtained from some of these individuals (see S2 and S3). At all localities we recorded advertisement calls of as many individuals possible, but only some were collected and preserved. Abbreviations for institutions are: Fundación Miguel Lillo, Instituto de Herpetología, Tucumán, Argentina (FML), zoological collection of the National University of Rio Cuarto (ECOALMUNRC) in Rio Cuarto, Argentina, Instituto y Museo de Ciencias Naturales (IMCNSJ), San Juan, Argentina, and the Zoologisches Forschungsmuseum „Alexander König“ (ZFMK) in Bonn, Germany.

10.7717/peerj.6480/supp-2 Supplemental Information 2 Allozyme allele frequencies in six nominal *Odontophrynus* taxa. Allozymes were scored with cellulose acetate electrophoresis and subsequently stained. The number of individuals scored is given in brackets.

10.7717/peerj.6480/supp-3 Supplemental Information 3 Samples of taxa used for molecular genetic analyses (partial sequences of the 16S rRNA gene), their geographic origins, voucher specimens, GenBank accession numbers, and original sources. Voucher abbreviations are AF (Laboratório de Citogenética de Vertebrados, Instituto de Biociências, Universidade de São Paulo, Brazil), CFBH (collection Célio F.B. Haddad, Universidade Estadual Paulista, Rio Claro, São Paulo, Brazil), CNP (Centro Nacional Patagónico, Chubut, Argentina), DB (Diego Baldo, Universidad Nacional de Misiones, Argentina), ECOALMUNRC (Zoological collection, National University of Rio Cuarto, Argentina), FSFL (field number of Felipe Sá Fortes Leite, Pontifícia Universidade Católica de Minas Gerais, Brazil), JC (field number of José Cassimiro, Universidade de São Paulo, Brazil), BB (Boris Blotto field series), MLP (Museo de La Plata, La Plata, Argentina), MVZ (Museum of Vertebrate Zoology, Berkeley, USA), MZUSP (Museu de Zoologia, Universidade de São Paulo, Brazil), QCAZ (Museo de Zoología de la Pontificia Universidad Católica del Ecuador), ZFMK (Zoologisches Forschungsmuseum Koenig, Bonn, Germany), ZSM (Zoologische Staatssammlung München, Germany), ZUFRJ (Departamento de Zoologia, Instituto de Biologia, Universidade Federal do Rio de Janeiro, Brazil).

10.7717/peerj.6480/supp-4 Supplemental Information 4 Size-related variation of dominant frequency in the advertisement call of the *O. occidentalis* group. Linear regression model of male SVL [mm] on dominant frequency [Hz]. For statistical details see text.

10.7717/peerj.6480/supp-5 Supplemental Information 5 Morphometric raw data.

10.7717/peerj.6480/supp-6 Supplemental Information 6 Bioacoustic raw data.

10.7717/peerj.6480/supp-7 Supplemental Information 7 Advertisement call of topotypical Odontophrynus achalensis. Call recorded at 21.3 °C water temperature.

10.7717/peerj.6480/supp-8 Supplemental Information 8 Advertisement call of Odontophrynus cf. achalensis. Call recorded at 16.8 °C water temperature.

10.7717/peerj.6480/supp-9 Supplemental Information 9 Advertisement call of Odontophrynus americanus. Call recorded at 19.5 °C water temperature.

10.7717/peerj.6480/supp-10 Supplemental Information 10 Advertisement call of topotypical Odontophrynus barrioi. Call recorded at 15.1 °C water temperature.

10.7717/peerj.6480/supp-11 Supplemental Information 11 Advertisement call of Odontophrynus cf. barrioi. Call recorded at 18.1 °C water temperature.

10.7717/peerj.6480/supp-12 Supplemental Information 12 Advertisement call of topotypical Odontophrynus cordobae. Call recorded at 19.7 °C water temperature.

10.7717/peerj.6480/supp-13 Supplemental Information 13 Advertisement call of topotypical Odontophrynus lavillai. Call recorded at 24.3 °C water temperature.

10.7717/peerj.6480/supp-14 Supplemental Information 14 Advertisement call of Odontophrynus occidentalis. Call recorded at 17.5 °C water temperature.

## Additional Information and Declarations

### Field Study Permissions

The following information was supplied relating to field study approvals (i.e., approving body and any reference numbers): The Córdoba Environment Agency (A.C.A.S.E.), Environmental Secretary of Córdoba Government (A01-2013), authorized our study and issued research and collecting permits.

### DNA Deposition

The following information was supplied regarding the deposition of DNA sequences: Sequences are available in GenBank. Accession numbers and details on voucher specimens used for sequencing are available in Table S3.

### Data Availability

The following information was supplied regarding data availability: The raw data are available in the Supplemental Files.
