# Supplementary material for: Integrative taxonomic reassessment of Odontophrynus populations in Argentina and phylogenetic relationships within Odontophrynidae (Anura)
Source: PeerJ. 2019 Feb 25;7:e6480. doi: 10.7717/peerj.6480 (PMC6394351; doi:10.7717/peerj.6480)
Supplement: Supplemental Information 1 — All specimens were examined for morphometric analyses. Tissue samples used for allozyme and barcoding sequences were obtained from some of these individuals (see S2 and S3). At all localities we recorded advertisement calls of as many individuals possible, but only some were collected and preserved. Abbreviations for institutions are: Fundación Miguel Lillo, Instituto de Herpetología, Tucumán, Argentina (FML), zoological collection of the National University of Rio Cuarto (ECOALMUNRC) in Rio Cuarto, Argentina, Instituto y Museo de Ciencias Naturales (IMCNSJ), San Juan, Argentina, and the Zoologisches Forschungsmuseum „Alexander König“ (ZFMK) in Bonn, Germany. [file peerj-07-6480-s001.docx]

| **Nominal taxon** | **Localities** | **Collection numbers** |
| --- | --- | --- |
| *Odontophrynus achalensis* | Córdoba Province: Arroyo La Ciénaga, Pampa de Achala, Sierra de Achala (31°36´05´´S, 64°52´17´´W, 2,149 m asl); Est. Los Tabaquillos (32º 25´28,41´´S, 64º 55´10´´W, 2050m asl). | ECOALMUNRC 173-178,  ZFMK 80917-80926 |
| *Odontophrynus* cf.  *achalensis* | San Luis Province: La Carolina, Coronel Pringles (32°50´17´´S, 66°06´19´´W, 1,965m asl). | ECOALMUNRC 158, 339-345,  FML 10220-10221, 15316-15318,  ZFMK 80954-80956 |
| *Odontophrynus americanus* | Buenos Aires Province: Villa Maquehua, Chivilcoy (35° 05´ 14.0´´S 59° 47´ 06.3´´W, 49m asl).  Córdoba Province: Achiras, Sierras de Comechingones (31°21´01´´S, 64°35´58´´W, 854m asl); Baretto (33°21´00´´S, 63°17´59´´W, 146m asl); Km 619, National Road·#8, 432 m asl; Km 624, National Road·#8, 459m asl; Km 657, National Road #8, 526m asl; La Escondida (32º 40´ 19,32´´S, 64º 31´ 12,82´´W, 697m asl); Parque Sarmiento, Rio Cuarto (33°06´21´´S, 64°20´02´´W, 458m asl); Piedra Blanca (33° 01´ 15.3´´ S64° 44´ 27.3´´W, 651m asl); Punilla (33°07´53´´S, 65°05´10´´W, 851m asl). | ECOALMUNRC 60-62, 90-91, 103-107, 142-146, 161-166, 192, 207-219, 352, 354, 356,  ZFMK 80893-80913 |
| *Odontophrynus barrioi* | La Rioja Province: Aguadita Springs, 30 km N Famatina (29°31´58´´, S 68°31´04´´W, 2,200m asl);  Catamarca Province: Río El Carrizal, Condor Huasi, (27° 29´ 51.4´´S, 67° 06´ 36.1´´W, 2,051m asl). | ECOALMUNRC 112-117, 284-286,  FML 03699, 04554, 04587, 03699-1/-7,  ZFMK 80916 |
| *Odontophrynus* cf. *barrioi* | San Juan Province: Aguada del Molle, Sierra Pie de Palo, Angaco (31°23´36´´S, 67°59´50´´W, 1,854m asl); Huerta de Guachi, Jachal (30° 01´ 06.7´´S, 68° 44´ 28.6´´W, 1,742m asl). | ECOALMUNRC 224-226, 276-282  FML 15320-15325,  IMCNSJ 6000-6001,  ZFMK 80957-80958 |
| *Odontophrynus cordobae* | Córdoba Province: Athos Pampa (SW); Berrotarán (32° 24´ 03.5´´S, 64° 24´ 01.4´´W, 618 m asl); Arroyo El Sauce, Villa General Belgrano (31°58’21´´S, 64°32’03´´W, 730m asl); Arroyo El Nogal Cañada del Sauce (32° 22´ 18.0 ´´S, 64° 38´ 21.2´´W, 705m asl); Rio de los Sauces (32°31´52´´S, 64°35´02´´W, 726m asl); Río Santa Rosa, Santa Rosa de Calamuchita (32°04´07´´S, 64°32´29´´W, 594m asl); San Clemente (SW); Tanti (31°21´01´´S, 64°35´58´´W, 885m asl). | ECOALMUNRC 87, 147-157, 194-205, 245-248,  FML 10222-10226,  ZFMK 80933-80939 |
| *Odontophrynus lavillai* | Santiago del Estero Province: Arroyo Casa del Tigre, Villa de la Punta (28°22´19´´ S, 64°48´06´´ W,430m asl); Monte Quemado, Copo, (25° 42´ 02.5´´S, 62° 42´ 44.7´´W, 223m asl.  Salta Province: Finca Los Colorados, Anta, 360m asl; Pocitos, San José de Pocitos, 500m asl; Finca San Javier, Anta, 462m asl. | ECOALMUNRC 179-184, 187-190,  FML 02794-1/–4, 03758, 03580-1/ -3, 03580-2, 04911, 04915, 04922-1/-2, 05305-1, 05879,06594  ZFMK 80952-80953 |
| *Odontophrynus occidentalis* | Córdoba Province: Achiras, south Sierra de Comechingones (31°21´01´´S, 64°35´58´´W,854m asl); Alpa Corral (32°41´53´´S, 64°43´24´´W, 864m asl); Arroyo El Sauce, Villa General Belgrano (31°58’21´´S, 64°32’03´´W, 730m asl); Las Albahacas, south Sierras de Comechingones (32°54´00´´S, 64°47´00´´W, 690m asl); Rodeo viejo (32° 21´ 52.0´´S, 64° 40´ 26.2´´W, 856m asl); San Clemente (31°42´50´´S, 64°37´16´´W, 943m asl).  San Luis Province: El Trapiche (33° 04´ 07.4´´S, 66° 04´ 03.3´´W, 1,124m asl).  Neuquén Province: Rio Negro (exact site unknown). | ECOALMUNRC 82, 160, 169-172, 220-223, 311-316,  FML 10227,  ZFMK 80940-80946, 80948 |
