# Supplementary material for: Integrative taxonomic reassessment of Odontophrynus populations in Argentina and phylogenetic relationships within Odontophrynidae (Anura)
Source: PeerJ. 2019 Feb 25;7:e6480. doi: 10.7717/peerj.6480 (PMC6394351; doi:10.7717/peerj.6480)
Supplement: Supplemental Information 2 — Allozymes were scored with cellulose acetate electrophoresis and subsequently stained. The number of individuals scored is given in brackets. [file peerj-07-6480-s002.docx]

| Locus + allele | | *O. americanus*  (n=45) | *O. cordobae*  (n=28) | *O. lavillai*  (n=12) | *O. occidentalis*  (n=17) | *O. achalensis* (n=16) | *O.* cf. *achalensis*  (n=6) | *O. barrioi*  (n=9) | *O.* cf. *barrioi*  (n=14) |
| --- | --- | --- | --- | --- | --- | --- | --- | --- | --- |
| cAAT | a | - | - | 0.083 | - | - | - | - | - |
|  | b | - | - | 0.917 | 0.059 | - | - | - | 0.214 |
|  | c | 0.978 | 0.964 | - | 0.941 | 1.000 | 1.000 | 1.000 | 0.786 |
|  | d | 0.022 | 0.036 | - | - | - | - | - | - |
| mAAT | | 1.000 | 1.000 | 1.000 | 1.000 | 1.000 | 1.000 | 1.000 | 1.000 |
| EST | a | - | 0.036 | - | - | - | - | 0.500 | 0.536 |
|  | b | 0.860 | 0.893 | - | 0.413 | 0.500 | 0.583 | 0.111 | - |
|  | c | 0.140 | 0.071 | 1.000 | 0.558 | 0.469 | 0.417 | 0.389 | 0.464 |
|  | d | - | - | - | 0.029 | 0.031 | - | - | - |
| G3PD | a | 0.011 | 0.038 | - | 0.029 | - | - | - | - |
|  | b | 0.989 | 0.962 | 1.000 | 0.883 | 0.656 | 1.000 | 0.889 | 1.000 |
|  | c | - | - | - | 0.088 | 0.344 | - | 0.111 | - |
| GPI | a | 0.106 | 0.500 | - | 0.029 | - | - | - | - |
|  | b | 0.805 | 0.481 | 1.000 | 0.088 | - | - | - | - |
|  | c | 0.089 | 0.019 | - | 0.883 | 1.000 | 0.833 | 1.000 | 1.000 |
|  | d | - | - | - | - | - | 0.167 | - | - |
| cIDH | a | 0.100 | - | - | - | - | - | - | - |
|  | b | 0.900 | 1.000 | 1.000 | 0.118 | 0.063 | 1.000 | - | 0.250 |
|  | c | - | - | - | 0.812 | 0.937 | - | 1.000 | 0.750 |
| mIDH | a | 1.000 | 1.000 | 1.000 | 1.000 | 1.000 | 1.000 | - | - |
|  | b | - | - | - | - | - | - | 1.000 | 1.000 |
| LDH | a | 0.040 | 0.036 | 0.091 | - | - | - | - | - |
|  | b | 0.099 | 0.071 | 0.636 | 0.324 | - | - | 0.111 | 1.000 |
|  | c | 0.861 | 0.903 | 0.273 | 0.676 | 0.938 | 1.000 | 0.889 | - |
|  | d | - | - | - | - | 0.062 | - | - | - |
| ME | a | 0.093 | 0.130 | 0.167 | 0.344 | 0.200 | 0.250 | - | 0.643 |
|  | b | 0.622 | 0.348 | 0.666 | 0.250 | 0.334 | - | - | 0.035 |
|  | c | 0.215 | 0.457 | - | 0.094 | 0.233 | 0.167 | 1.000 | 0.179 |
|  | d | 0.070 | 0.065 | 0.167 | 0.312 | 0.233 | 0.583 | - | 0.143 |
| cMDH | a | - | - | - | - | - | - | 0.333 | - |
|  | b | 1.000 | 1.000 | 1.000 | 1.000 | 1.000 | 1.000 | 0.667 | 1.000 |
| mMDH | | 1.000 | 1.000 | 1.000 | 1.000 | 1.000 | 1.000 | 1.000 | 1.000 |
| 6PGD | a | 0.911 | 1.000 | 1.000 | 1.000 | 1.000 | 1.000 | 1.000 | 0.214 |
|  | b | 0.089 | - | - | - | - | - | - | 0.786 |
| cPGM | a | 0.116 | 0.019 | - | - | - | - | - | - |
|  | b | 0.732 | 0.962 | 1.000 | 0.235 | 0.344 | - | 0.167 | - |
|  | c | 0.152 | 0.019 | - | 0.765 | 0.625 | 1.000 | 0.833 | 0.583 |
|  | d | - | - | - | - | 0.031 | - | - | 0.417 |
| mPGM | a | 1.000 | 1.000 | 1.000 | 1.000 | 0.938 | 0.833 | 1.000 | 1.000 |
|  | b | - | - | - | - | 0.062 | 0.167 | - | - |
