# Supplementary material for: Integrative taxonomic reassessment of Odontophrynus populations in Argentina and phylogenetic relationships within Odontophrynidae (Anura)
Source: PeerJ. 2019 Feb 25;7:e6480. doi: 10.7717/peerj.6480 (PMC6394351; doi:10.7717/peerj.6480)
Supplement: Supplemental Information 3 — Voucher abbreviations are AF (Laboratório de Citogenética de Vertebrados, Instituto de Biociências, Universidade de São Paulo, Brazil), CFBH (collection Célio F.B. Haddad, Universidade Estadual Paulista, Rio Claro, São Paulo, Brazil), CNP (Centro Nacional Patagónico, Chubut, Argentina), DB (Diego Baldo, Universidad Nacional de Misiones, Argentina), ECOALMUNRC (Zoological collection, National University of Rio Cuarto, Argentina), FSFL (field number of Felipe Sá Fortes Leite, Pontifícia Universidade Católica de Minas Gerais, Brazil), JC (field number of José Cassimiro, Universidade de São Paulo, Brazil), BB (Boris Blotto field series), MLP (Museo de La Plata, La Plata, Argentina), MVZ (Museum of Vertebrate Zoology, Berkeley, USA), MZUSP (Museu de Zoologia, Universidade de São Paulo, Brazil), QCAZ (Museo de Zoología de la Pontificia Universidad Católica del Ecuador), ZFMK (Zoologisches Forschungsmuseum Koenig, Bonn, Germany), ZSM (Zoologische Staatssammlung München, Germany), ZUFRJ (Departamento de Zoologia, Instituto de Biologia, Universidade Federal do Rio de Janeiro, Brazil). [file peerj-07-6480-s003.docx]

| **Nominal Taxon** | **Locality** | **Voucher** | **GenBank #** | **Original source** |  |
| --- | --- | --- | --- | --- | --- |
| *Odontophrynus achalensis* | | Pampa de Achala, Sierra de Cordoba, Cordoba, Argentina  Est. Los Tabaquillos | ZSM 733/2000 | DQ283247 | Frost et al. (2006) |
|  |  |  | MLP 4766 | KP295642 | Faivovich et al. (2014) |
|  |  |  | BB 1324 | DQ283248 | Frost et al. (2006) |
|  |  |  | ECOALMUNRC 178 | MK131160 | this study |
| *Odontophrynus americanus* | | Poços de Caldas, Minas Gerais, Brazil | AF 665 | FJ685686 | Amaro et al. (2009) |
|  |  | Airuoca, Minas Gerais, Brazil | CFBHT08133 | KU495398 | Lyra et al. (2017) |
|  |  | Rio Grande, Rio Grande do Sul, Brazil | CFBHE00334 | KU495402 | Lyra et al. (2017) |
|  |  | Aracoiaba da Serra, Sao Paulo, Brazil | CFBHT08110 | KU495401 | Lyra et al. (2017) |
|  |  | Irai, Santa Catarina, Brazil | CFBHT08305 | KU495400 | Lyra et al. (2017) |
|  |  | Teodoro Sampaio, Sao Paulo, Brazil | CFBHT09582 | KU495399 | Lyra et al. (2017) |
|  |  | Escobar, Loma Verde, Buenos Aires, Argentina | JF1891 | AY843704 | Faivovich et al. (2005) |
| *Odontophrynus barrioi* | | Aguadita, Sierra de Famatina, La Rioja, Argentina | ZFMK 80916 | MK131161 | this study |
|  |  | Arroyo el Carrizal, Belén, Catamarca, Argentina | ECOALMUNRC 284 | MK131162 | this study |
| *Odontophrynus* cf. *barrioi* | | Aguada del Molle, Sierra Pie de Palo, San Juan, Argentina | ECOALMUNRC 228 | MK131163 | this study |
| *Odontophrynus carvalhoi* | | Mucugê, Bahia, Brazil | JC 1224 | FJ685687 | Amaro et al. (2009) |
| *Odontophrynus cordobae* | | Berrotaran, Cordoba, Argentina | ZFMK 80932 | MK131164 | this study |
|  |  | Villa General Belgrano, Cordoba, Argentina | ZFMK 80934 | MK131165 | this study |
|  |  |  | ZFMK 80936 | MK131166 | this study |
| *Odontophrynus cultripes* | | Varginha, Minas Gerais, Brazil | FSFL 875 | FJ685688 | Amaro et al. (2009) |
|  |  | Campo Limpo de Goias, Goias, Brazil | CFBHT02188 | KU495403 | Lyra et al. (2017) |
| *Odontophrynus lavillai* | | Villa la Punta, Santiago del Estero, Argentina | ZFMK 80952 | MK131167 | this study |
|  |  |  | ZFMK 80953 | MK131168 | this study |
| *Odontophrynus occidentalis* | | Rio Negro, Neuquen, Argentina | ZFMK 95407 | MK131169 | this study |
|  |  | Lujan de Cuyo, Finca Vistalba, Mendoza, Argentina | MVZ:Herp 145207 | JX564880 | Zhang et al. (2013) |
|  |  |  | MVZ:Herp 145210 | FJ882744 | Van Bocxlaer et al. (2009) |
| *Macrogenioglottus alipioi* | | Caucaia do Alto, Cotia, São Paulo, Brazil | AF 1607 | FJ685685 | Amaro et al. (2009) |
|  |  | Serra do Teimoso, Jussari, Bahia, Brazil | AF 919 | FJ685684 | Amaro et al. (2009) |
|  |  | Urucuca, São Paulo, Brazil | CFBH 12929 | KC593360 | Fouquet et al. (2013) |
| *Proceratophrys appendiculata* | | São Sebastião, São Paulo, Brazil | MZUSP 135333 | FJ685690 | Amaro et al. (2009) |
| *Proceratophrys avelinoi* | | Misiones, Argentina | JF 1948 | DQ283039 | Frost et al. (2006) |
| *Proceratophrys bigibbosa* | | Misiones, Argentina | DB 2313 | FJ 685692 | Amaro et al. (2009) |
| *Proceratophrys boiei* | | Serra da Cantareira, São Paulo, Brazil | AF 1587 | FJ685692 | Amaro et al. (2009) |
| *Proceratophrys concavitympanum* | | Usina Hidrelétrica de Lajeado,  Palmas, Tocantins, Brazil | AF 1094 | FJ685694 | Amaro et al. (2009) |
| *Proceratophrys cristiceps* | | Parque Nacional da Serra das  Confusões, Piauí, Brazil | AF 887 | FJ685695 | Amaro et al. (2009) |
| *Proceratophrys cururu* | | Cardeal Mota, Minas Gerais, Brazil | FSFL 580 | FJ685696 | Amaro et al. (2009) |
| *Proceratophrys laticeps* | | Reserva Natural da Vale do Rio Doce, Linhares, Espírito Santo, Brazil | AF 1900 | FJ685698 | Amaro et al. (2009) |
| *Proceratophrys melanopogon* | | Maringá, Itatiaia, Rio de Janeiro, Brazil | CFBH 5755 | FJ685699 | Amaro et al. (2009) |
| *Proceratophrys minuta* | | Miguel Calmon, Bahia, Brazil | AF2309 | JX982966 | Teixeira et al. (2009) |
| *Proceratophrys moratoi* | | Itirapina, São Paulo, Brazil | CFBH 6515 | FJ685689 | Amaro et al. (2009) |
| *Proceratophrys redacta* | | Morro do Chapeu, Bahia, Brazil | MTR 22579 | JX982967 | Teixeira et al. (2009) |
| *Proceratophrys renalis* | | Brejo Madre de Deus, Pernambuco, Brazil | ZUFR J8682 | FJ685700 | Amaro et al. (2009) |
| *Proceratophrys schirchi* | | São Lourenço, Santa Teresa, Espírito Santo, Brazil | 371 | FJ685701 | Amaro et al. (2009) |
| *Proceratophrys tupinamba* | | Ilha Grande, Rio de Janeiro, Brazil | MNRJ 54541 | KF214158 | Dias et al. (2013) |
| *Rhinella marina* | | Puerto Cayo, Manabi, Ecuador | QCAZ 50698 | KR012643 | Dos Santos et al. (2015) |
| *Pleurodema somuncurensis* | | El Rincon, Meseta Somuncura, Rio Negro, Argentina | CNP A404 | JQ937203 | Faivovich et al. (2012) |
| *Ceratophrys cornuta* | | Cusco Amazonico, Madre de Dios, Peru | KU 202561 | AY326014 | Darst and Cannatella (2004) |
