# Supplementary figures and images for: Integrative taxonomic reassessment of Odontophrynus populations in Argentina and phylogenetic relationships within Odontophrynidae (Anura)
Source: PeerJ. 2019 Feb 25;7:e6480. doi: 10.7717/peerj.6480 (PMC6394351; doi:10.7717/peerj.6480)

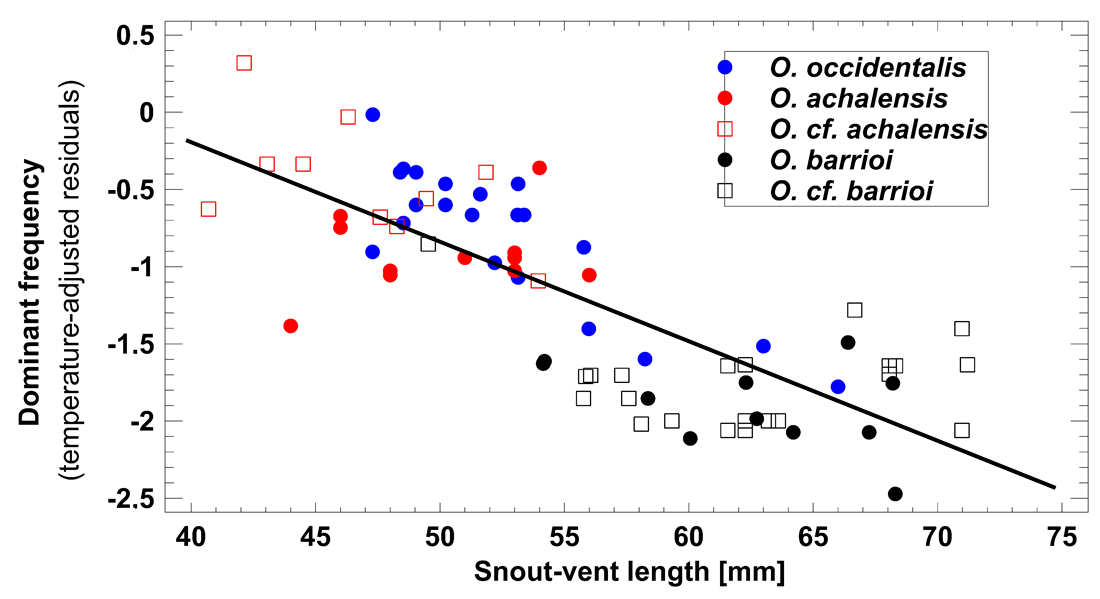

Supplement: Supplemental Information 4 — Linear regression model of male SVL [mm] on dominant frequency [Hz]. For statistical details see text. [file peerj-07-6480-s004.png]
